# Supplementary material for: Silica-associated proteins from hexactinellid sponges support an alternative evolutionary scenario for biomineralization in Porifera
Source: Nat Commun. 2024 Jan 7;15:181. doi: 10.1038/s41467-023-44226-7 (PMC10772126; doi:10.1038/s41467-023-44226-7)
Supplement: Supplementary file 1 — Supplementary Information [file 41467_2023_44226_MOESM1_ESM.pdf]

# Supplementary Information

**Silica-associated proteins from hexactinellid sponges support an alternative evolutionary scenario for biomineralization in Porifera**

Katsuhiko Shimizu[1*], Michika Nishi[2], Yuto Sakate[2], Haruka Kawanami[3], Tomohiro Bito[3], Jiro Arima[3], Laia Leria[4], Manuel Maldonado[4**]

[1]Platform for Community-based Research and Education, Tottori University, 4-101, Koyama-cho Minami Tottori 680-8550, Japan
[2]Division of Agricultural Science, Graduate studies of Sustainability Science, Tottori University Graduate School, 4-101, Koyama-cho Minami, Tottori 680-8553, Japan
[3]Department of Life Environmental Agriculture, Faculty of Agriculture, Tottori University, 4-101, Koyama-cho Minami, Tottori 680-8553, Japan
[4]Sponge Ecobiology and Biotechnology Group, Center for Advanced Studies of Blanes (CEAB), CSIC, Blanes 17300, Spain

*Correspondence: kshimizu@tottori-u.ac.jp
** Correspondence: maldonado@ceab.csic.es

## Index of Supplementary Information

**Supplementary Text**
Supplementary Text-1. Biotechnological derivations of sponge silicification.
Supplementary Text-2. On the presence of chitin and actin in sponge spicules.
Supplementary Text-3. On the absence of actin in SDS-PAGE and Western blot.
Supplementary Text-4. Systematic allocation of hexactinellid genomes and transcriptomes.
Supplementary Text-5. Hexaxilin-like sequences.
Supplementary Text-6. Metallo-beta lactamase fold proteins.
Supplementary Text-7. Homology of axial filaments.
Supplementary Text-8. Perisilin-like sequences.
Supplementary Text-9. Extracellular silicification in Demospongiae.
Supplementary Text-10. On the origin of Homoscleromorpha

**Supplementary Figures**
Supplementary Fig. 1. SDS-PAGE and Western blot analyses.
Supplementary Fig. 2. Effects of a loose outermost silica layer on fluorochrome distribution.
Supplementary Fig. 3. Conserved Cysteine and Zn2+- binding positions in hexactinellid hexaxilins.
Supplementary Fig. 4. Alignments of hexaxilin-like sequences of Porifera.
Supplementary Fig. 5. Maximum likelihood phylogenetic tree of hexaxilins.
Supplementary Fig. 6. SWISS-MODEL templates for inferring secondary protein structure.
Supplementary Fig. 7. 3D models of hexaxilin-1 of *Euplectella curvistellata.*
Supplementary Fig. 8. 3D models of hexaxilin-1 of *Vazella pourtalesii.*
Supplementary Fig. 9. Alignments of perisilin-like sequences of Porifera.
Supplementary Fig. 10. Maximum likelihood phylogenetic tree of perisilins.
Supplementary Fig. 11. Conserved cysteine and histidine positions in hexactinellid perisilin proteins.
Supplementary Fig. 12. 3D models of perisilin-1 of *Vazella pourtalesii.*
Supplementary Fig. 13. Maximum likelihood phylogenetic tree of glassins.

Supplementary Fig. 14. Conserved histidine and aspartic acid positions in hexactinellid glassin proteins
Supplementary Fig. 15. 3D models of glassin of *Euplectella curvistellata.*
Supplementary Fig. 16. Expression pattern of actin in *Vazella pourtalesii*.

**Supplementary Tables**
Supplementary Table S1. Phobius predictions for signal peptides in hexaxilin, perisilin and glassin.

**Supplementary References**
References 1 to 61.

# Supplementary Text

**Supplementary Text-1. Biotechnological derivations of sponge silicification**.

Mastering the biogeochemistry of sponge silicification has potential interest in many more areas than indicated in the introductory main text, because silica is nowadays one of the most industrially important inorganic compounds. It is utilized in many technological applications, not only as solid silica but also in aqueous sol-gel solutions[1]. Here we summarize briefly some examples related to promising biological and biomedical applications.

Mesoporous silica nanoparticles, sometime synthesized by manipulating demosponge silicateins and diatom silaffins, are being widely assayed in drug delivery and related biomedical applications[2,3]. The biomimetic and bioinspired silicas are also used to immobilize and stabilize sensitive biomolecules, such as enzymes, antibodies, and catalytic proteins[4]. Biomimetic silicas are being tested to encapsulate living cells and viruses, as well. The latter process, applied to viruses, aims —among other things—to confer thermostability to viral vaccines[5]. Silica-based materials enzymatically generated using sponge-derived silicatein proteins are being considered to generate 3D-bioprinted scaffolds[6] for bone regeneration in dental implants and other situations needing rapid osteogenesis[7–9]. However, although, biosilica-based materials are quite promising in bone biomaterial formation, the technology for their biomedical application is still limited due to challenges to increase the control of the silica distribution in the biomaterials[10,11]. Thus, the better the natural biosilicification processes are known, the larger the possibilities to success in their biotechnological harnessing.

**Supplementary Text -2. On the presence of chitin and actin in hexactinellid spicules.**
Apart from silicatein and glassin, other organic components have been reported from inside sponge spicules, such as the amino polysaccharide chitin and the protein actin. To date, chitin has been identified in a variety of 21 marine and three fresh-water sponge species[12], but rarely incorporated into the silica pieces. It has never been found in the silica of demosponges and homoscleromorphs, occurring only in some hexactinellid spicules[13–17]. There is agreement that chitin does not actively participate in the process of silica deposition[13–15] and there is experimental evidence that it neither improves the resistance of sponge silica to dissolve in seawater[18]. Indeed, the role of chitin in hexactinellid spicules remains quite enigmatic. We suspect that, in the case of the species *Ijimalophus hawaiicus,* chitin could provide mechanical aid for spicule bending. This sponge grows on a stalk as high as 50 cm, formed by hundreds of needle-like, giant (up to 50 cm long) spicules. The spicule holdfast protrudes from the base of the sponge body downwards to anchor the animal to the soft sediments[19]. These spicules contain 15.3 ± 1.3 µg chitin per mg of silica, which occurs in the form of nanometer-thick layers intercalated between the concentric silica layers[17]. The body of the sponge is cup-like, with the edge of the cup flexed outwards, for the sponge to works as a sort of parachute designed to capture currents, what facilitates filter

feeding[20]. In order for these sponges to readjust their body orientation and take better advantages of changes in the intensity and direction of local currents, the spicules of the stalk have to twist and bend without breaking, following the changes in the direction and intensity of currents. That can only happen if the concentric silica layers of the spicule, instead of being fused to each other (as it normally happens), are separated by a chitin interlayer that would allow two adjacent silica layers to slide relative to each other for spicule twisting and bending. Thus, the occurrence of chitin within only some spicule types of only some hexactinellids (i.e., not being a common or widespread feature in the group) likely results from singular species adaptions, with different function in each case.

The situation is different for actin, a widespread protein common in both the cytoplasm and the nucleus of animal cells, which has also been reported to occur in association with the axial filament of Demospongiae and Hexactinellida[21] and, more recently, Homoscleromorpha[22]. The exact role of the F-actin detected in sponge spicules remains under discussion. F-actin is known not to induce silica deposition itself. The absence of hydroxyproline (3-Hyp and 4-Hyp) residues responsible for the attachment of silicic acid to the protein excludes it as a plausible direct template for silicification[21]. Actin was rather hypothesized to act as a putative template around which the units of the actual silicifying proteins of the axial filaments (i.e., silicatein) would assemble[21]. Given the capacity of F-actin to produce bifurcating and dendritic filaments, it has also been proposed that it might be involved in patterning the 3-D architecture of spicules[21]. However, such a suggestion needs to be further examined, as the sequence of F-actin is highly conserved not only among classes of Porifera but also across the metazoan tree, while the 3D shape of sponge spicules varies drastically between orders, families and even related genera.

**Supplementary Text-3. On the absence of actin in SDS-PAGE and Western blot analyses.**
Our SDS-PAGE and Western blot analyses of the silica extracts revealed well-marked bands of glassin, perisilin and hexaxilin, as corresponding to abundant protein components in the studied spicules (Supplementary Fig. 1a, c, e). Additionally, some other — but much fainter— protein bands showed up, all of them with a molecular weight higher than 70 kDa (red arrows in Supplementary Fig. 1e). Sequence information on these minority bands could not be obtained because their abundance in the gels is consistently low. Since actin is a 42 kDa protein, it is ruled out that any of those minority protein bands with high molecular weight could be actin. The question arises as to why our approach aimed at revealing the silicifying proteins does not pick up the presence of actin in the hexactinellid silica, unlike that reported by Ehrlich et al.[21].

There are a number of potential reasons, including important differences between our methodological approach and that of Ehrlich et al.[21]. First, we performed a more aggressive cleaning of the outer organic matter of the spicules by immersion in bleach and then in concentrated $HNO_3$/H2$SO_4$ (1:4) at room temperature, whereas Ehrlich et al. immersed the spicules in only 70% $HNO_3$ at room temperature for 3 days. Our more aggressive cleaning makes it easier for bleach and/or acids to infiltrate between the loose silica layers characterizing many of the hexactinellid spicules[18] or entering through cracks in the silica that lead to the axial canal, posing in greater risk the integrity of some of the proteins of the axial filament. We also conducted a much more aggressive digestion of the silica by complete immersion of the acid-cleaned spicules in HF/$NH_4F$ (2M:8M) for 2-3 days, whereas Ehrlich and co-workers[21] used only 10% HF (~5 M) droplets falling on the spicules for 7h to 10h. Another major difference in the approach is that we used the extracts from the total silica digestion, whereas Ehrlich and co-workers used the extracts from only axial filaments previously released from the silica. The process of concentration of the proteins in the extracts was also markedly different. Ehrlich et al.[21] were only able to retrieve actin when the soluble extracts of the axial filament were precipitated with ice cold acetone, incubated at -20°C and centrifuged at 10,000 g for 10 seconds to obtain protein pellets. The need for such precipitation steps suggests that actin was in low concentration in those filaments. We did not perform such a precipitation step. For protein concentration in the soluble

fraction, we used ultrafiltration. Theoretically, ultrafiltration would also concentrate actin, but, in practice, actin could also have been lost from our soluble fraction by selectively binding to the ultrafiltration membrane or by precipitating during ultrafiltration. There are also differences in the electrophoresis approach. We used NOVEX NuPAGE system, in which proteins always run at neutral pH conditions. In contrast, proteins are subjected to alkaline pH during electrophoresis in the conventional SDS-PAGE used by Ehrlich et al.[21]. The NuPAGE system gives better resolution than the conventional system and, in fact, glassin does not appear in the conventional SDS-PAGE system. In this regard, we do not know whether actin might also behave unusually in the NuPAGE system. In summary, due to the aforementioned methodological differences, the absence of actin in our gels cannot be used to decide whether this protein is actually absent or present in the silica of *E. curvistellata* and *V. pourtalesii*.

Interestingly, we identified three actin genes in the transcriptome of *V. pourtalesii*. One of them (*Actin-1*) has five isoforms, two of which (α and β) became drastically upregulated under high dSi concentrations (Supplementary Data 3-4, Supplementary Fig. 16). Again, our approach cannot decide whether such upregulation derives from an involvement of actin in the process of spicule production or it is resulting from the countless other cell processes in which this protein participates.

**Supplementary Text-4. Systematic allocation of hexactinellid genomes and transcriptomes**. The search of genomes and transcriptomes in open sources indicated that data are available currently for only seven species of Hexactinellida, representing various lineages (Fig. 1d), as it follows: 1) In the subclass Hexasterophora and order Lyssacinosida, the rosellids *Vazella pourtalesii, Rossella fibulata*, and *Sympagella nux*, the euplectellid *Euplectella curvistellata*, and the leupsacid *Oopsacas minuta*; 2) In the subclass Hexasterophora and order Sceptrulophora, the aphrocallistid *Aphrocallistes vastus*; 3) In the subclass Amphidiscophora and order Amphidiscosida, the hyalonematid *Hyalonema populiferum*.

**Supplementary Text-5. Hexaxilin-like sequences.**
NCBI and EukProt hexaxilin blastp revealed hexaxilin-like sequences in Porifera and members of four other metazoan phyla, in five marine protists, and a diversity of bacteria but no Archaea (Supplementary Text-6).

Hexaxilin-like sequences were identified in sponges from the classes Hexactinellida, Calcarea and Demospongiae (Supplementary Fig. 4), but, in all cases, with very low identities (25-29%) and E-values larger than $6.0e^{-31}$, which casted doubts about function preservation and, in some cases, even about true sequence homology. In the class Demospongiae, occurrence of transcripts or genes is limited to subclass Heteroscleromorpha. The species expressing hexaxilin-like genes among subclass Heteroscleromorpha were *Amphimedon queenslandica, Haliclona amboinensis, Xestospongia testudinaria, Kirpatrickia variolosa, Latrunculia apicalis,* and *Ephydatia muelleri*. *Hexaxilin*-like transcripts were not found in Heteroscleromorpha sponges *Haliclona tubifera, Petrosia ficiformis, Crella elegans, Stylissa carteri,* and *Pseudospongosorites suberitoide*. Likewise, *hexaxilin*-like transcripts were found neither in the Dictyoceratida sponge *Ircinia fasciculate*, which does not produce silica spicule, nor in the Verongimorpha sponge *Chondrilla nucula*¸ which produces silica spicules. In addition, *Leucosolenia complicata* and *Sycon ciliatum*, both belonging to the class Calcarea (i.e., sponges producing spicule of calcium carbonate but not of silica) contained hexaxilin-like transcripts. In the class Homoscleromorpha, *hexaxilin*-like sequences were not present, neither in silicifying species, such as *Corticium candelabrum*¸ nor in aspiculate species, such as *Oscarella carmela* and *Oscarella lobularis*. Interestingly, the hexaxilin-like sequences of Calcarea make independent, cohesive group that does not relate to hexaxilin-like sequences of either Hexactinellida or Demospongiae (Fig. 4). In contrast, hexaxilin-like sequences of Demospongiae make two independent groups, one related

to those of Hexactinellida and the other related to those of Aplicomplexa protists and non-sponge metazoans.

The metazoan sequences include three scyphozoan cnidarians (*Aurelia* sp., *Rhopilema esculentum*, *Sanderia malayensis*), three annelids (*Capitella teleta*, *Hormogaster samnitica, Sipunculus nudus*), a brachiopod (*Lingula anatina*), and a chordate tunicate (*Oikopleura dioica*). Most hexaxilin-like proteins of non-poriferan metazoans are expected to have diverged towards functions of MBL fold proteins unrelated to silicification. Nevertheless, it cannot be ruled out that, as more genomes are completed, new hexaxilin-like sequences or other proteins with silicifying activity will be identified in additional animal lineages. Besides polychaetes and brachiopods, there are other metazoans able to form structures that are partially silicified, such as teeth of copepods[23], limpets[24] and nudibranchs[25]. The molecular machineries behind silica deposition in non-poriferan metazoans are largely understudied.

**Supplementary Text-6. Metallo-beta lactamase fold (MBLf) proteins.**
Search for Conserved Domain Database[26] (CDD; https://www.ncbi.nlm.nih.gov/Structure/cdd/wrpsb.cgi) indicated that hexaxilins are related to COG2333, a zinc metal-dependent hydrolase with a metallo-beta-lactamase fold in the C-terminal domain of the DNA uptake channel protein ComEC. Bacterial protein MBE7455146, which shares the highest similarity (29%) with hexaxilins, is a MBLf metallo-hydrolase. MBLf metallo-hydrolases catalyze hydrolysis of substrates through one or two $Zn^{++}$ incorporated into conserved residues[27,28], which, in the hexaxilin sequences, show some level of aa substitution. Thus, hexaxilins probably have no capability to bind the metal ions needed to unfold the hydrolase activity. The lack of metal ions in hexaxilins might be responsible for hexactinellid spicules containing only small amounts of metal elements, including Fe, Zn, and Cu[29].

**Supplementary Text-7. Homology of axial filaments.**
The axial filament of demosponges is hexagonal or triangular in cross section[30], while that of hexactinellids it is square [31]. Earlier X-ray diffraction analyses had indicated differences between hexactinellids and demosponges in the arrangement and size of the protein molecules constituting their respective axial filaments[32,33]. Also, the axial filaments of demosponges had been described as consisting of closely contacting proteins, while those of hexactinellids would be composed by proteins leaving wide gaps in the arrangement, which silica fills in[34]. All these previously suggested differences now make new sense, after discovering that the main component of the axial filament is hexaxilin in Hexactinellida and silicatein in Demospongiae. Thus, the axial filaments of hexactinellids and demosponges, from the point of view of molecular biology, are not homologs, but analogous structures independently developed to perform similar functions during silicification.

**Supplementary Text-8. Perisilin-like sequences.**
Perisilins of Hexactinellida are loosely related (identity < 32%) to perisilin-like sequences of the CAP superfamily[35] occurring in bilaterians (Supplementary Data 5). The CAP family includes cysteine-rich secretory proteins, antigen 5 proteins, and pathogenesis-related proteins. More specifically, hexactinellid perisilins are related to cysteine-rich transcripts from the mollusk *Crassostrea virginica* and the echinoderm *Anneissia japonica,* which are in turn related to latisemin, the cystein-rich glycoprotein venom of *Laticauda* spp. sea snakes[36]. Perisilins are also loosely related to peptidase inhibitor proteins from mollusks, cephalochordates and fish (Supplementary Data 5), as well as to glioma pathogenesis-related protein 1 (GLIPR1), first identified from a human brain tumor (glioblastoma), which in turn is related to plant pathogenesis-related proteins[37]. Nearly all known CAP proteins are secreted and operate extracellularly[35], except GLIPR1 and GLIPR2, which are transmembrane[38].

**Supplementary Text-9. Extracellular silicification in Demospongiae.**
In demosponges, there is evidence that microscleres and megascleres of small size (up to 250-300 µm in length) can be produced entirely within the scleroecytes[39–41]. However, it is difficult to maintain that mm-long and cm-long spicules, which are orders of magnitude larger than sclerocyte cells, are finished intracellularly. Thus, it is long suspected[30,42] that, once large megascleres attain a certain size intracellularly, they are exocytosed by the sclerocyte to the mesohyl, where they experience further steps of extracellular silicification. These extracellular steps remain poorly understood. Some authors have suggested that the sclerocytes produce a rain of tiny exosome-like vesicles (silicasomes) that contain inactivated silicatein and dSi at high concentration[43–45]. Somehow, this cocktail becomes activated once the silicasomes contact the spicule surface to induce the extracellular of deposition silica. Other hypothesis suggests that the sclerocytes crawl on the exocytosed spicules while somehow releasing dSi at high concentration on the spicule surface, which would produce extracellular silicification without invoking any protein participation[46]. Both hypotheses remain disputed, each leaving important facts unexplained. Regarding the former hypothesis, it is worrying the fact that the hypothesized extracellular rain of silicasomes around the growing spicules cannot be regularly corroborated in routine TEM studies. Regarding the second hypothesis, some sort of extracellular protein or other organic compound need to be invoked to induce dSi polymerization and to spatially guide such an extracellular silica deposition. According to the former hypothesis an extracellular deposition of silicatein onto the surface of the growing spicules would somehow guide the extracellular silica condensation[47,48].

Silicatein is currently understood as the only silicifying protein in Demospongiae. However, other proteins, such as collagen, silintaphin and galectin, have been proposed to be related to the demosponge silica. Though these are not considered as silicifying proteins, they are suggested to interact somehow with silicatein during the silicification process. Silintaphin-1 would help the silicatein monomers to get spatially organized to construct the 3D axial filament, so that the core of the axial filament would be a filament of silintaphin that acts as a template that becomes subsequently covered by silicatein, which would then catalyze silica deposition[49]. Although silintaphin-1 is not a silicifying protein itself, in vitro incubations of both silicatein and silintaphin have shown higher activity in silica polymerization than incubations of just silicatein[49,50], probably because improved 3D structure of the in vitro axial filaments.

Galectins are lectin proteins that bind to galactose sugars and its derivatives. These proteins are widespread in metazoans and fungi and perform a broad variety of functions. They are soluble, occurring either at intracellular or extracellular locations and being particularly abundant extracellularly in the mesohyl of demosponges. There, galectins have been proposed to link to extracellular silicatein. The interaction would facilitate that silicatein can form layers on the surface of extracellular stages of developing spicules, which would account for successive waves of extracellular deposition of peripheral silica and spicule thickening[48,51].

**Supplementary Text-10. On the origin of Homoscleromorpha**
The Homoscleromorpha are a small class of Porifera that represents less than 1% of extant Porifera. Skeletally, these sponges are characterized by having siliceous skeletal pieces that are comparatively smaller (< 300µm) and less diversified in shape than those of Demospongiae and Hexactinellida[52,53]. The bulk of the fossil record of Homoscleromorpha is far younger than that of demosponges and hexactinellids, with common fossil in the Tertiary, the oldest unambiguous fossil spicules dating from the Upper Jurassic (161-145 mya)[54], and some less unambiguous skeletons from the Lower Carboniferous (360-299 mya)[55]. There is, however, a controversial re-interpretation that spicules of the Late-Ordovician (some 455-445 mya), hexactinellid-like sponge *Cyathuphycus laydelli*[56] could be similar to those of modern homosclerophorid, because they have an structure with marked concentric layers when sliced in cross section[57]. Such a proposal results arguable from the available evidence and it would also leave unexplained an enormous gap (100

to 250 million years) of fossil emptiness in the fossil record, between the occurrence of the putative Ordovician spicules and the following Carboniferous or Jurassic records.

In striking contrast with the bulk of the fossil record, molecular clocks estimate the origin of Homoscleromorpha in the Precambrian, that is, a few hundred million years before the oldest unambiguous Carboniferous-Jurassic fossils. Some of these molecular approaches propose that Homoscleromorpha would have diverged from Demospongiae some 862-712 mya[58]. Others propose that Homoscleromorpha did not diverge from Demospongiae but from Calcarea, some 900-700 mya[59]. In any case, both proposals agree with a Precambrian origin for Homoscleromorpha. It means that these sponges would have thrived in the Paleozoic oceans without a siliceous skeleton for a time period several hundred million years longer than those of Hexactinellida and Demospongiae, until the Carboniferous-Jurassic. During the Paleozoic and until the Jurassic, estimates from marine chert analyses suggests that dSi values in the global ocean were still notably high, that is, around 1,000 μM[60].

However, unlike in the case of Hexactinellida and Demosponges, the hypothesis that homoscleromorph skeletons are lacking from most of the Paleozoic fossil record because those sponges were not yet skeletonized gets troubled by a recent discovery. Experiments on dissolution of sponge silica have shown that the spicules of Homoscleromorpha dissolve at rates far faster than those of Hexactinellida and Demospongiae, what strongly suggests that they have much higher chances to dissolve before entering the sedimentary fossil record[18].

## Supplementary Figures

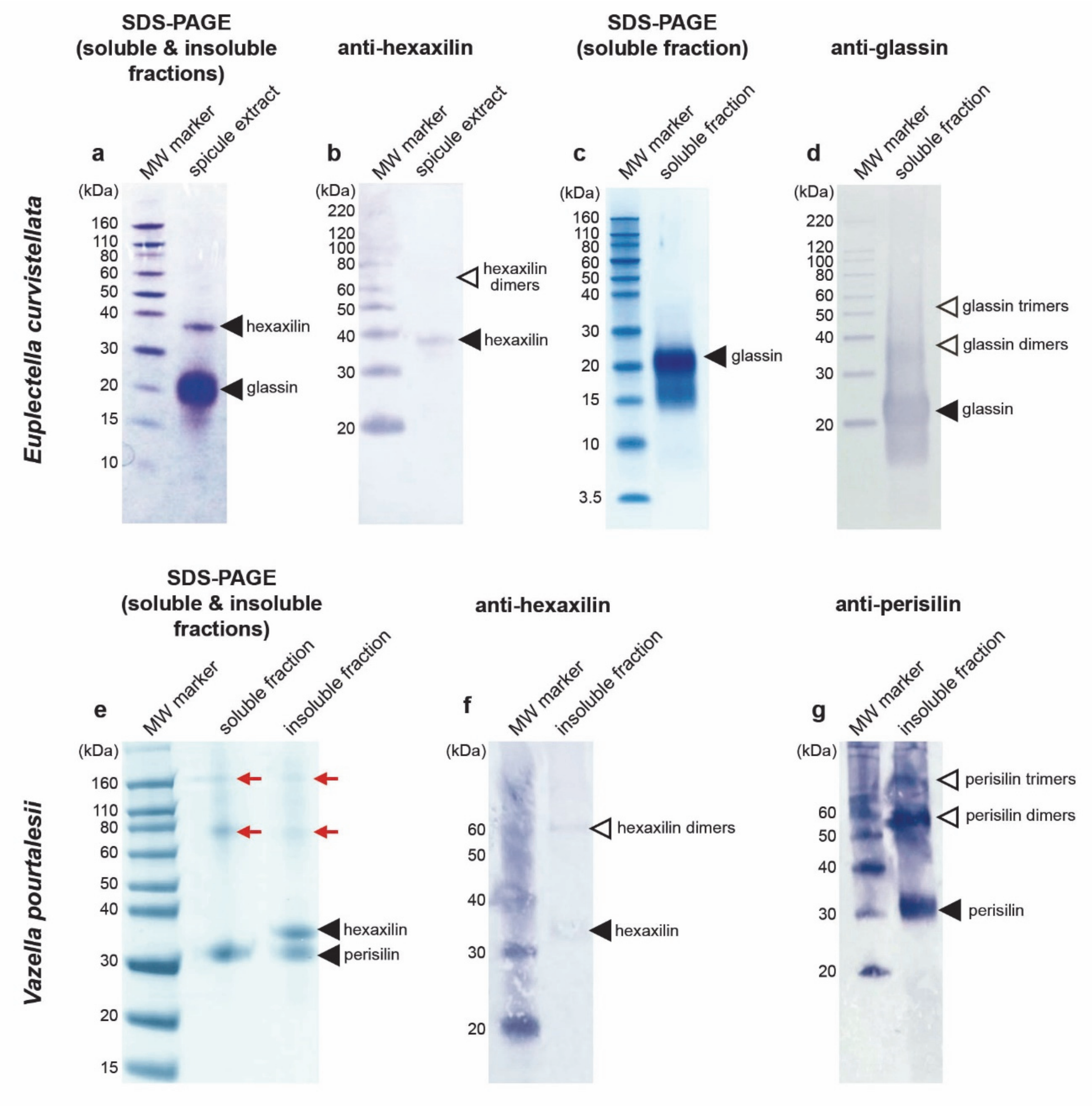


**Supplementary Fig. 1. SDS-PAGE and Western blot (Wb) analyses of extracts from hexactinellid silica.** SDS-PAGE analysis **(a, c)** of the combined water-soluble and water-insoluble spicule extracts of *Euplectella curvistellata*, along with Wb analyses of that combined extract using 1:1000 anti-hexaxilin antiserum **(b)** and of only the soluble fraction using 1:1000 anti-glassin antiserum **(d).** Note that the monomeric units of these proteins appear to self-polymerize easily, with the putative dimers and trimers being retained in faint bands (white arrow heads) of high molecular weight, which are also specifically tagged by the corresponding antibodies**.** SDS-PAGE analysis of the water-soluble and water-insoluble spicule extracts of *Vazella pourtalesii* **(e)**, along with Wb analyses of the insoluble extract treated with 1:100 anti-hexaxilin **(f)** and 1:100 anti-perisilin affinity-purified antibodies (**g**). Note the appearance of faint bands of putative dimers and trimers despite the use of reducing agents, bands that are specifically recognized by their respective antibodies. Bands corresponding to actin (42 kDa) do not occur in any of the three SDS-PAGE analyses **(a, c, e)**, while additional minor bands (red arrows) of unidentified proteins occur at molecular weighs higher than 70 kDa.

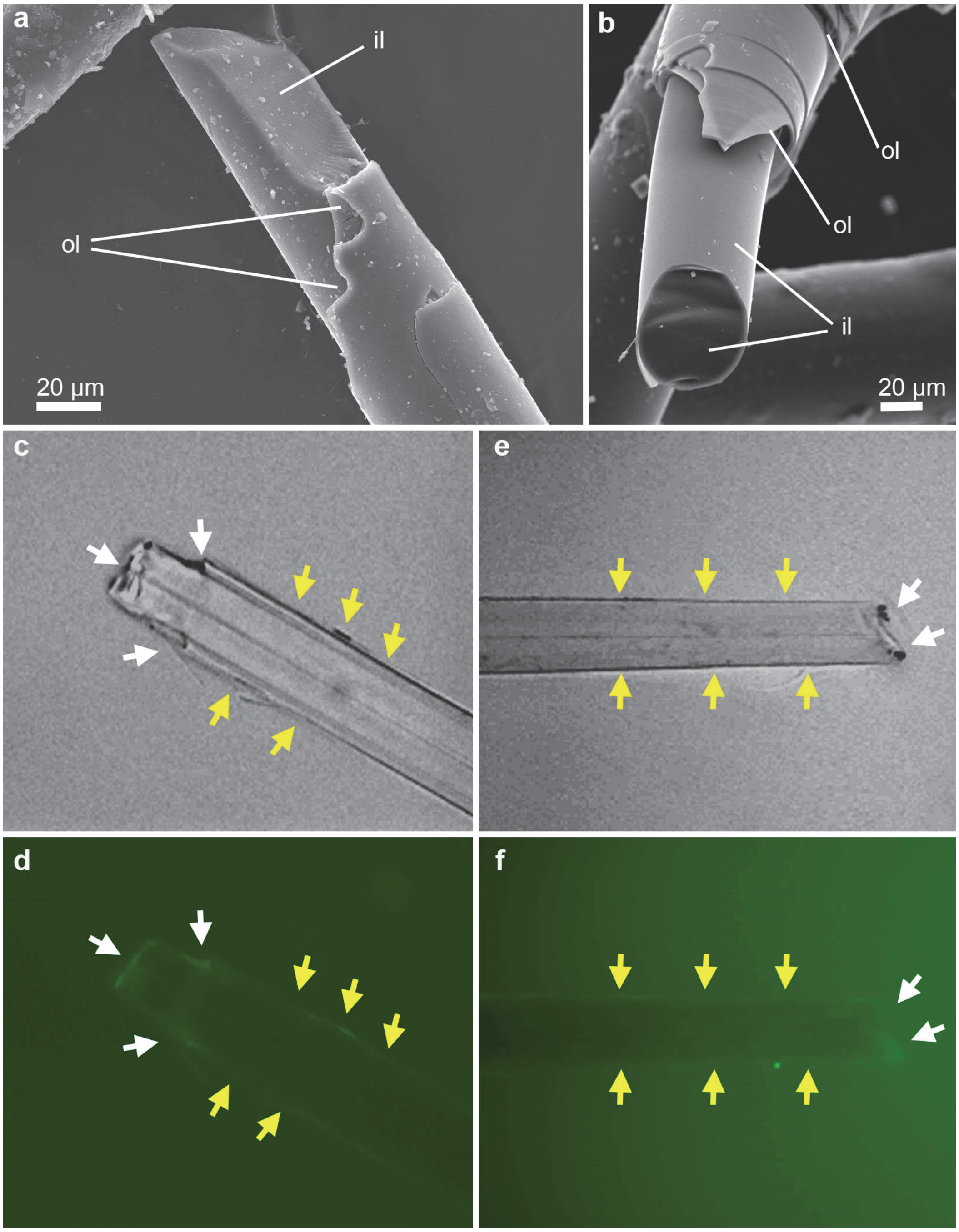


**Supplementary Fig. 2. Effects of a loose outermost silica layer on fluorochrome distribution. (a-b)** Scanning electron microcopy views of spicules of *Vazella pourtalesii* showing that the outermost layer of silica (ol) consists of sublayers that are loosely attached to each other and relative to the internal silica core (il) of the spicule. Such a feature is known to be more pronounced in *V. pourtalesii* and other rossellids than in another hexactinellid groups[18,61]. **(c-d)** Spicule of *V. pourtalesii* incubated with pre-immune serum against hexaxilin. Note in the bright field image (c) the occurrence of portions with broken silica (white arrows) and loose peripheral silica (yellow arrows), both of which adsorb the fluorochrome by capillarity, which subsequently cannot be completely removed even after several rinsing steps, causing a very week staining that might lead to erroneous interpretations about the specificity of the antibodies for the axial filament. **(e-f)** Spicule of *Euplectella curvistellata* incubated with pre-immune serum against hexaxilin. Note that the adsorption effect by the peripheral silica is less pronounced, because the layers are less loose than in *V. pourtalesii.* These controls with pre-immune serum indicate that, as shown in Fig. 2 of main text, the designed anti-hexaxilin antibodies label with specificity only the protein in the axial filament but no protein constituents of the peripheral silica.

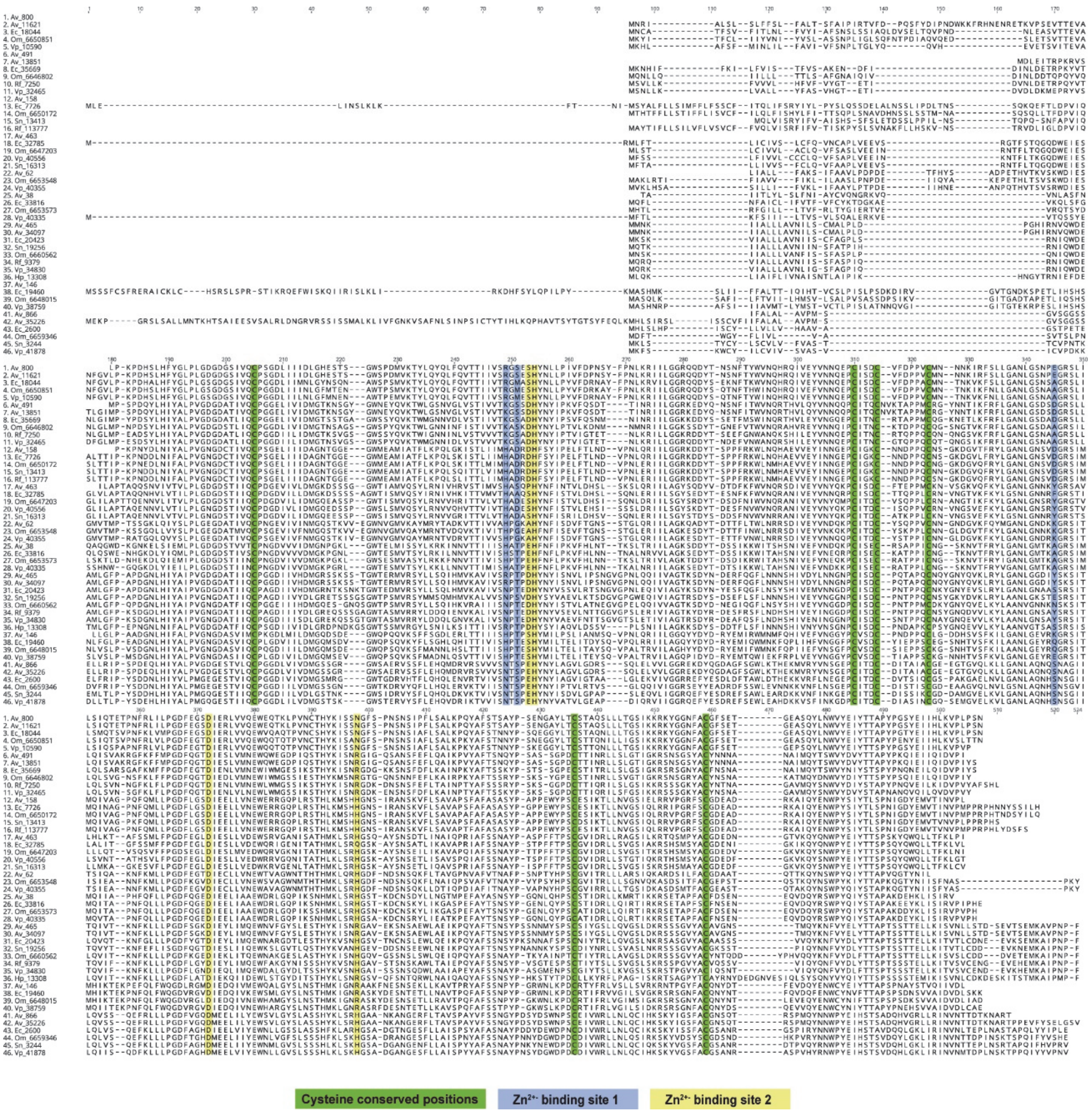


**Supplementary Fig. 3. Conserved Cysteine and non-conserved $Zn^{2+}$-binding positions in hexactinellid hexaxilins**. All amino acid sequences were aligned using MAFFT-online. Sequence codes as in Fig. 4, Supplementary Fig. 5 and Supplementary Data 1. Alignment data are available in fasta format in Source Data Supplementary Fig. 3. Ec: *Euplectella curvistellata*, Av: *Aphrocallistes vastus,* Sn: *Sympagella nux*, Om: *Oopsacas minuta*, Rf: *Rossella fibulata*, Vp: *Vazella pourtalesii* and Hp: *Hyalonema populiferum.*

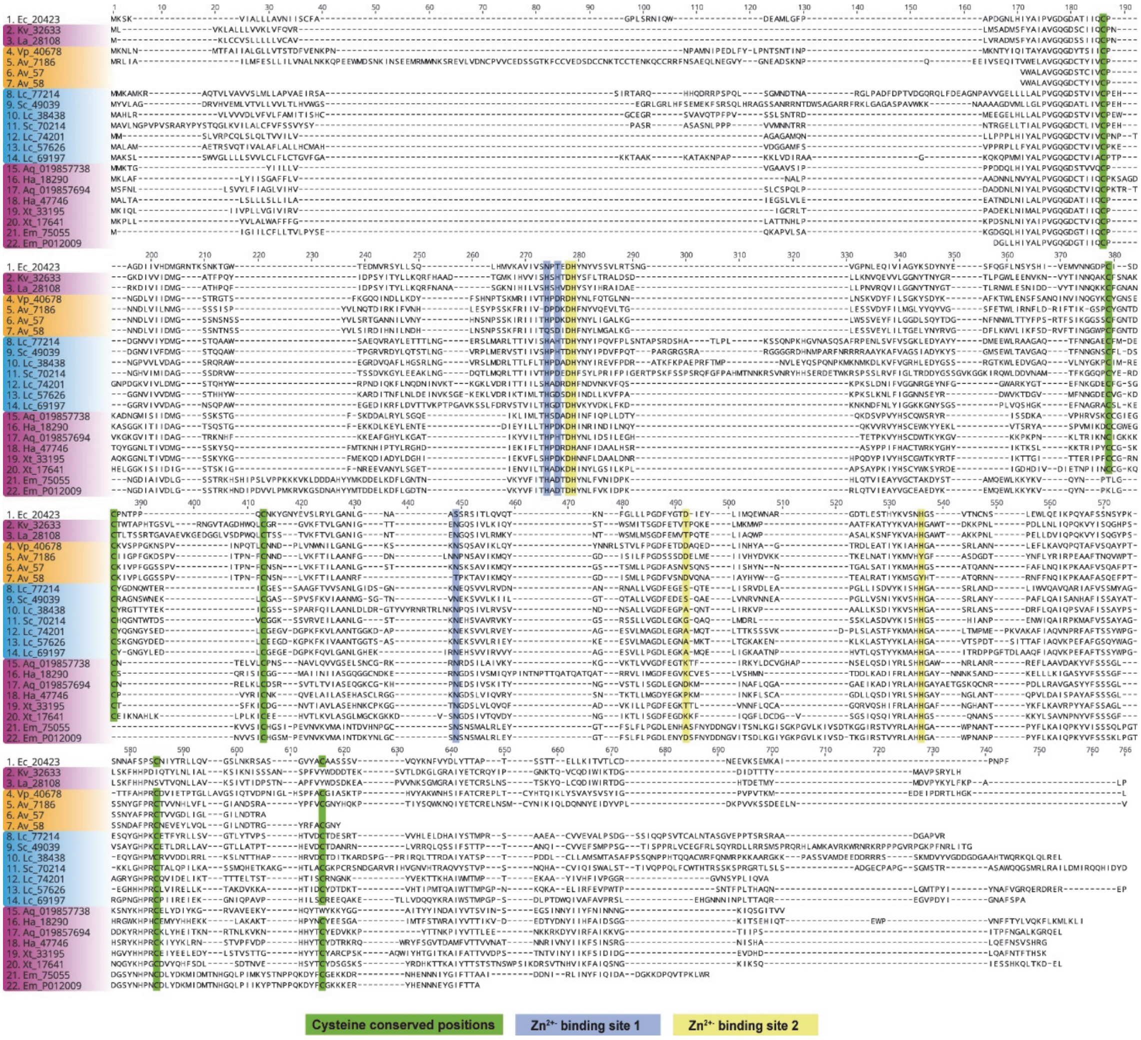


**Supplementary Fig. 4. Alignments of hexaxilin-like sequences of Porifera.** Conserved cysteine positions and predicted $Zn^{2}$+ binding positions in hexaxilin-like proteins of Calcarea (blue), Demospongiae (purple) and Hexactinellida (orange) are compared to Hexaxilin-1 of *Euplectella curvistellata* (Ec_20423). The sequences were aligned using MAFFT-online. Sequence codes are as in Fig. 4 and Supplementary Data 1. Alignment data is available in fasta format in Source Data Supplementary Fig. 4. All sequences of Calcarea and Hexactinellida have six cysteine positions conserved, whereas, among Demospongiae, variability occurs in *Kirpatrickia variolosa*, *Latrunculia apicalis*, *Amphimedon queenslandica* and *Ephydatia muelleri*. Three of the seven putative $Zn^{2+}$- binding sites are conserved (positions 278, 279 and 536) and the four others show amino acids different from those in hexaxilin-1 of *E. curvistellata*. Note that several hexaxilin-like sequences of Calcarea, Demospongiae and Hexactinellida show long amino acid insertions compared to hexaxilin-1 of *E. curvistellata*, which may change protein conformation and/or function.

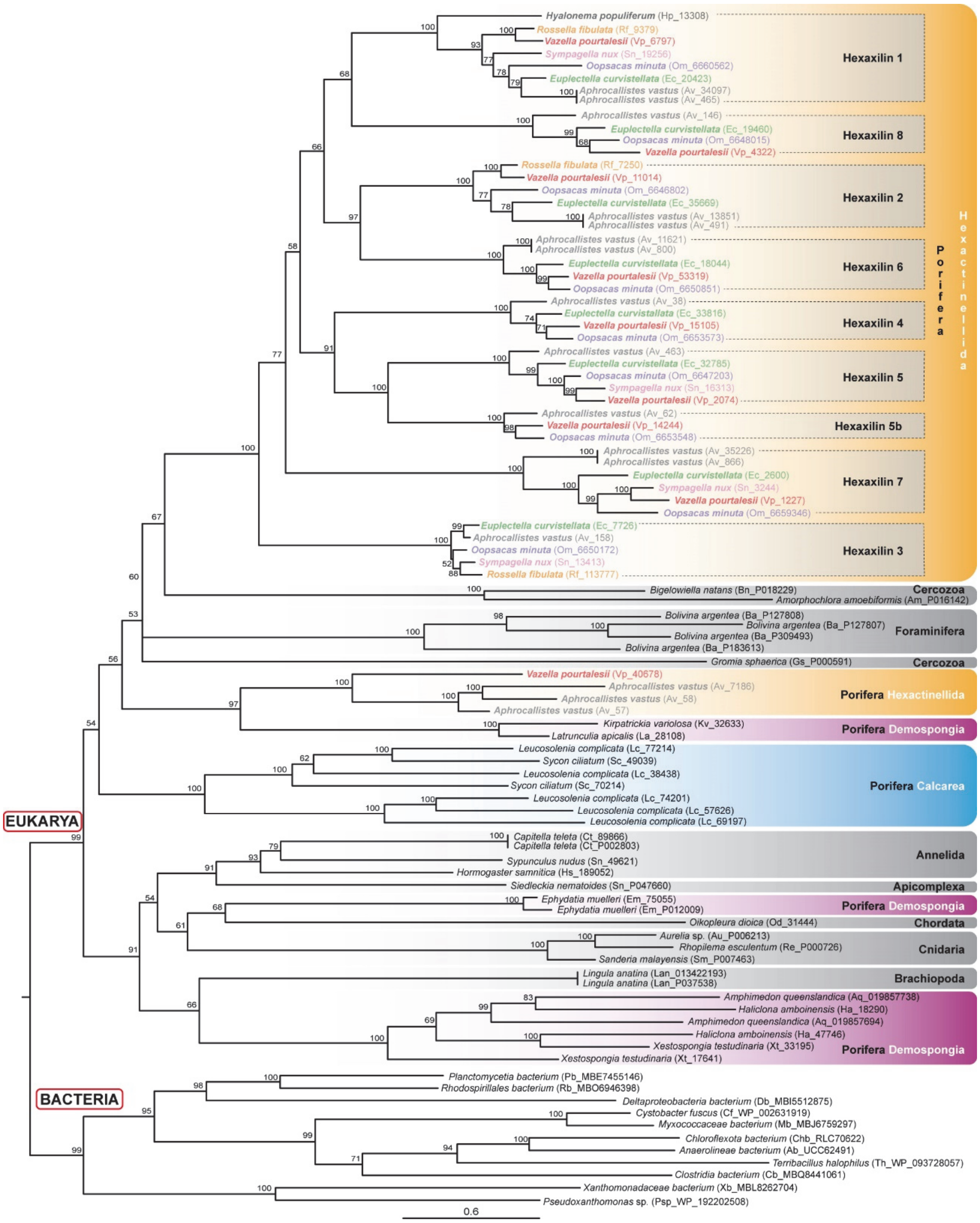


**Supplementary Fig. 5. Maximum Likelihood phylogenetic tree of hexaxilins obtained with IQ-TREE.** Included sequences correspond to all blast hits for hexaxilin-1 of *E. curvistellata* (sequence Ec_20423) with a bit score larger than 50 (Supplementary Data 1). Each hexactinellid species is represented by a different colour. Scale bar represents 0.5 amino acid substitutions per site. Bootstrap values are given at each node. Alignment data is available in fasta format in Source Data Fig. 4.

**a**

```
Ec_hxx1  MKSKVIALLLAVNIISCFAGPLSRNIQWDEAMLGFPAPDGNLHIYAIPVGDG--DATIIQCPAGDIIVHDMGRNTKSNK-
1wra.1.A ---------------------------------------SGNKIHFINVQEGGSDAIILES-NGHFAMVDTGEDYDFPDG

Ec_hxx1  ----TGWT-----------EDMVRSYLLSQ-LHMVKAVIVSNPTEDHYNYVSSVLRTSNGVGPNLEQIVIAGYKSDY---
1wra.1.A SDSRYPWREGIETSYKHVLTDRVFRRLKELSVQKLDFILVTHTHSDHIGNVDELLSTY-----PVDRVYLKKYSDSRITN

Ec_hxx1  ---------NYESFQGFLNSYSHIV-EMVNNGDPCISDCPNTPPQCNKYGNYEVSLRYLGANL----------GNAASSR
1wra.1.A SERLWDNLYGYDKVLQTATETGVSVIQNITQGDAH-----------FQFG--DMDIQLYNYENETDSSGELKKIWDDNSN

Ec_hxx1  SITLQVQTKNFGLLLPGDFYGTDIEYLIMQEWNARGDTLESTHYKVSNHG-SVTNCNSLEWLQEIKPQYAFSSNSYPKSN
1wra.1.A SLISVVKVNGKKIYLGGDLDNVH-GAE--DKYG--PLIGKVDLMKFNHHHDTNKS-NTKDFIKNLSPSLIVQTSD--SLP

Ec_hxx1  NAFSPSCNIYTRLLQVGSLNKRSA---SGVYACAASSSVVQYKNFVYDLYTTAPTSSTTELLKITVTLCDNEEVKSEMKA
1wra.1.A WKNGVDSEYVNWLKERGIERINAASKDYDATVFD----------------------------------------------

Ec_hxx1  IPNPF
1wra.1.A -----
```

**b**

```
Vp_hxx1  MQRKVIALLLAVNLIGSFAGPIQRNIQWDEAMLGFPKSDGNLHIYAIPVGNG--DATIIQCPGGDIIISDLGREKQSSGT
1wra.1.A ---------------------------------------SGNKIHFINVQEGGSDAIILES-NGHFAMVDTGEDYDFPDG

Vp_hxx1  -----GW-----------TASMVRRYLDDQ-LGNVKALIVSNPTEDHYNYVAEVFNTTSGVGTSLETIVIAGTRSDYR--
1wra.1.A SDSRYPWREGIETSYKHVLTDRVFRRLKELSVQKLDFILVTHTHSDHIGNVDELLSTY-----PVDRVYLKKYSDSRITN

Vp_hxx1  -------DEAFQSFLNDH---SHI-VENINGGNPCISDCSNTPPQCNAYGNQEVVLKYLAANLG----------NSAYSR
1wra.1.A SERLWDNLYGYDKVLQTATETGVSVIQNITQGDA-----------HFQF--GDMDIQLYNYENETDSSGELKKIWDDNSN

Vp_hxx1  SITTQVIFKNFKLLLPGDFLGNDIEQLIMQEWDALGYDLTSSHYKVSNHGAISSSNSQDWLAAIAPEYAFSSNAYPASGM
1wra.1.A SLISVVKVNGKKIYLGGDLD-NVHGAE--DKY--GPLIGKVDLMKFNHHHDTNKSNTKDFIKNLSPSLIVQTSDSL-PWK

Vp_hxx1  ENPSCGIYTSLLKVGSLSKRST-----PGVYACIQSNTVIQYQNFVYDLFTTSPTSSSTELLKITVSVCENDEVKHEMKA
1wra.1.A NGVDSEYVNWLKERGIERINAASKDYDATVFDIRKDGFVNIS--------------------------------------

Vp_hxx1  IPNPF
1wra.1.A -----
```

**c**

```
Ec_glsin HHHSHAPPSPPTVPPHSHPLPPHTHATVPHTHATVPHTHATHTHHTHTTPSPGLFGDEIDVQFELASDELQRPGHHGHHG
1wht.1.A --------------------------------------------------------------------------------

Ec_glsin HHDHDHDHDHDHDHGKHDHHGKHGKHGKHDHHDHHHSHAPPSPPTVPPHSHPLPPHTHATVPHTHATVPHTHATHTHHTH
1wht.1.A --------------------------------------------------------------------------------

Ec_glsin TTPSPGLFGDEIQGHGNNHDHGNHDHHGNNHDHPDHHTTSGPHPHSPLDVIDLDSADESNFEDEPLEMFSNPESWTKSVN
1wht.1.A ------------------------------------------------------------------GLVLNEYRWNKVAN

Ec_glsin VVYFDF--AAGFARSASN-----------SFDFALIIEKLCRLHPELIGAEVYVGGKNFGAYFASLAFEDIRSSNETEIT
1wht.1.A VLFLDSPAGVGFSYTNTSSDIYTSGDNRTAHDSYAFLAKWFERFPHYKYRDFYIAGESYAGHYVPELSQLVHRSKNPVIN

Ec_glsin AKGFILSLSAPEDNDKYDSLEDISLIMNGAEFKTEFGLDETDIWDPYLSDIFEQDQYTNDQLTAFTENPAGKFSTVIYGR
1wht.1.A LKGFMVGNG-----------------------------------------------------------------------

Ec_glsin DVTADDLKFE
1wht.1.A ----------
```

**d**

```
Vp_pslin1  MKGIIAILLLSFFSIAFAMPGLTQQQKRQIVDEINMERRFISAYAANMREVRWSECLQKLAGNFLLQCNPSGSSNAMRNA
3q2u.1.A.  --------------------IENEDFIKDCVRIHNKFRSEVKPTASDMLYMTWDPALAQIAKAWASNCQFSHNTRLKPP-

Vp_pslin1  QAIGARCESSRVGETIFNVSH--FANYNPIAVWAAQKEYYDND--QCDEYDCSDYLQLVDSGSFAVGCEIMDESMKCG--
3q2u.1.A.  --HKLHPNFTSLGENIWTGSVPIFSVSSAITNWYDEIQDYDFKTRICK-KVCGHYTQVVWADSYKVGCAVQFCPKVSGFD

Vp_pslin1  --RPGMTAVCYYALDGT----TSTAGIPCSKCEGEWKQCNNGLCSHRRTNGQGGNSRSNQSNGNQGNHGNHELPRPHGNH
3q2u.1.A   ALSNGAHFICNYGPGGNYPTWPYKRGATCSACPNN-DKCLDNLCVNRQR-------------------------------

Vp_pslin1  GNHELPTPHGNHGNHELPTPHGHQHRFHPQNDENKHH
3q2u.1.A   -------------------------------------
```

**Supplementary Fig. 6. Alignments of the spicule proteins characterized in the present study and the SWISS-MODEL templates used to infer their 3D secondary structure**. **a,** Hexaxilin-1 of *E. curvistellata* and **b,** hexaxilin-1 of *V. pourtalesii* aligned with 1wra.1.A (teichoic acid phosphorylcholine esterase/choline binding protein E, cbpE) as a template, **c,** Glassin of *E. curvistellata* aligned with 1wht.1.A (serine carboxypeptidase II) as a template, **d,** Perisilin-1α of *V. pourtalesii* aligned with 3q2u.1.A (glioma pathogenesis-related protein 1) as a template.

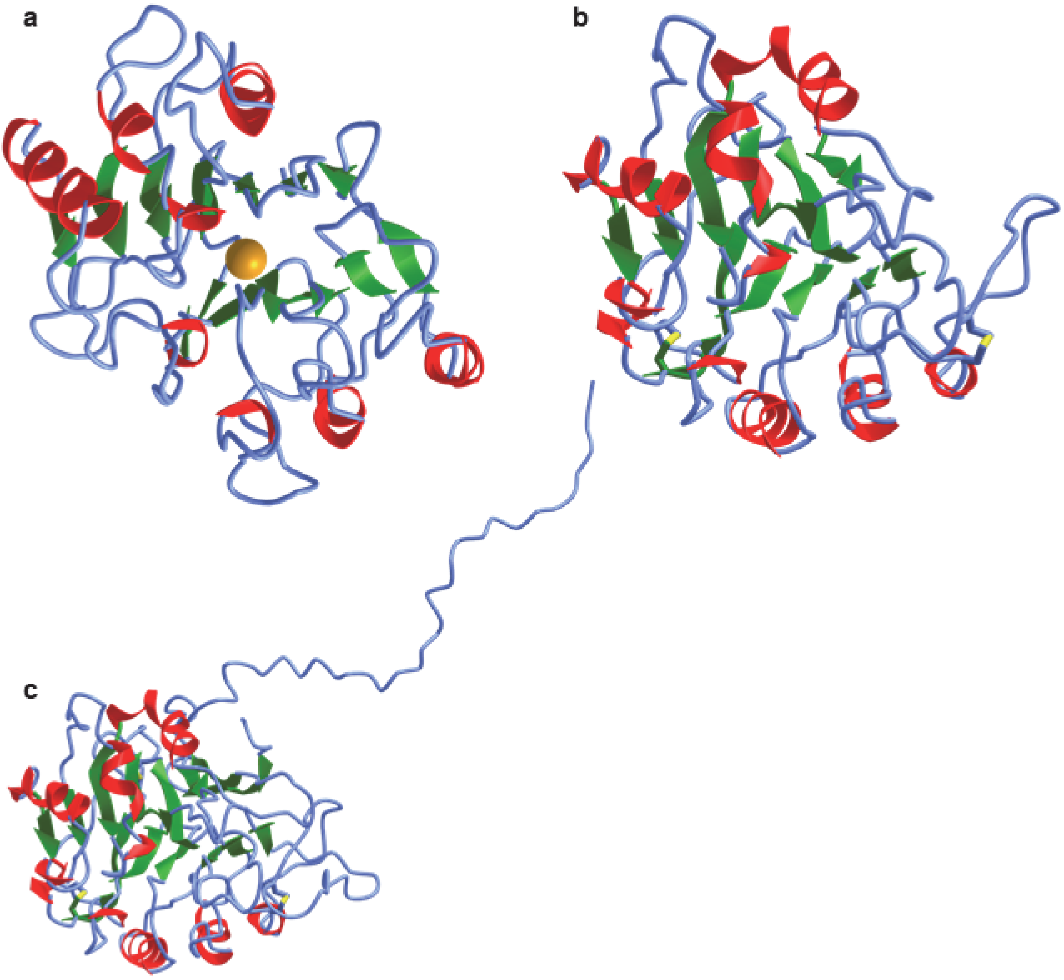


**Supplementary Fig. 7. 3D models of hexaxilin-1 of *Euplectella curvistellata*.** Models were built using 1wra.1.A (teichoic acid phosphorylcholine esterase/choline binding protein E, cbpE) as a template for SWISS-MODEL **(a)** or Alphafold2 **(b, c)**. Model in image **a** represents amino acids 41-308 in the sequence, because the template covered only such a region (see Supplementary Fig. 6). The same peptide sequence (i.e., 41-308 aa) was selected for comparing 3D inference in image **b** using Alphafold2 (**b**). Three-D structure inferred by Alphafold2 on the basis of the entire aa sequence (**c**). Note that regions unavailable in the template are basically predicted by Alphafold2 as with random structure. Sequences expected to form alpha-helices and beta-sheets are colored in red and green, respectively.

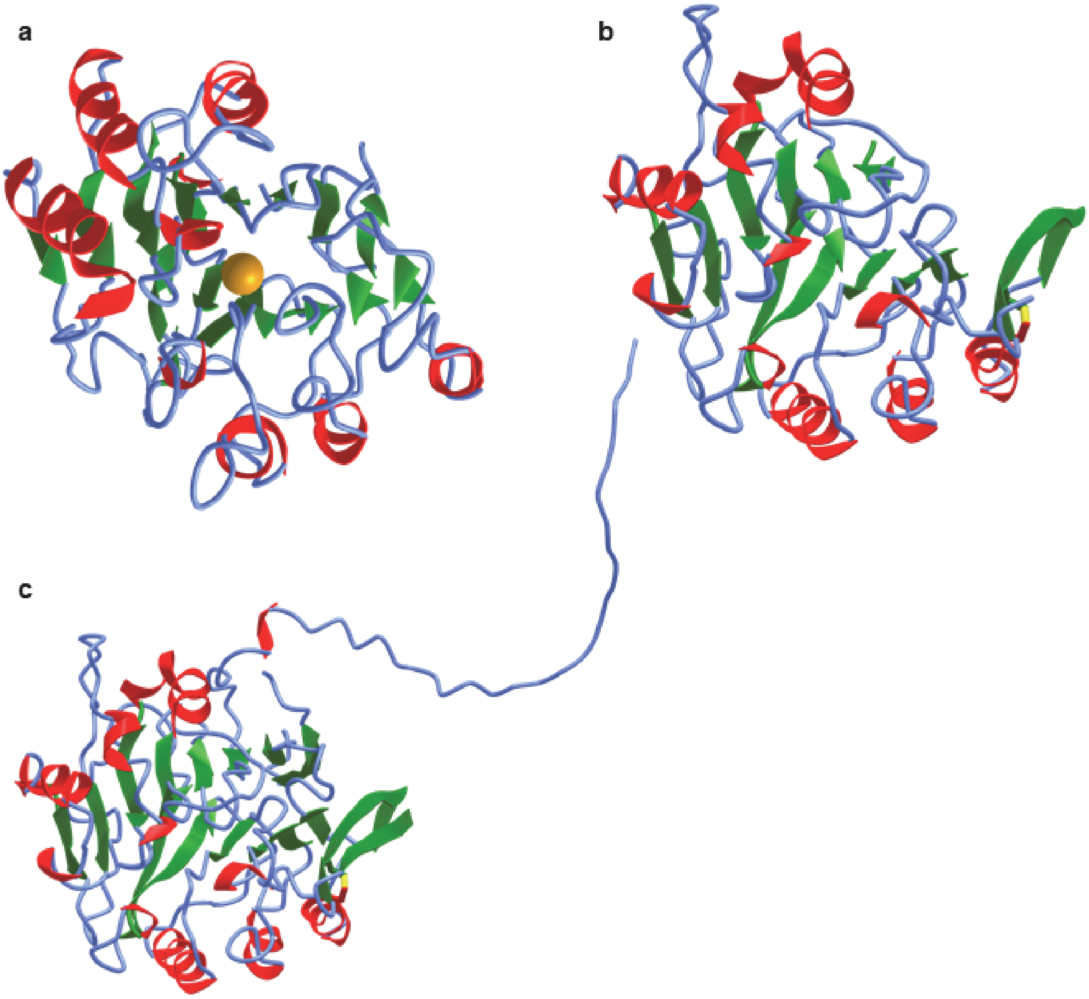


**Supplementary Fig. 8. 3D models of hexaxilin-1 of *Vazella pourtalesii*.** Models were built using 1wra.1.A (teichoic acid phosphorylcholine esterase/choline binding protein E, cbpE) as a template for SWISS-MODEL **(a)** or Alphafold2 **(b, c)**. Model in image **a** represents amino acids 41-315 in the sequence, because the template covered only such a region (see Supplementary Fig. 6). The same peptide sequence (i.e., 41-315 aa) was selected for comparing 3D inference in image **b** using Alphafold2 (**b**). Three-D structure inferred by Alphafold2 on the basis of the entire aa sequence (**c**). Note that regions unavailable in the template are basically predicted by Alphafold2 as with random structure. Sequences expected to form alpha-helices and beta-sheets are colored in red and green, respectively.

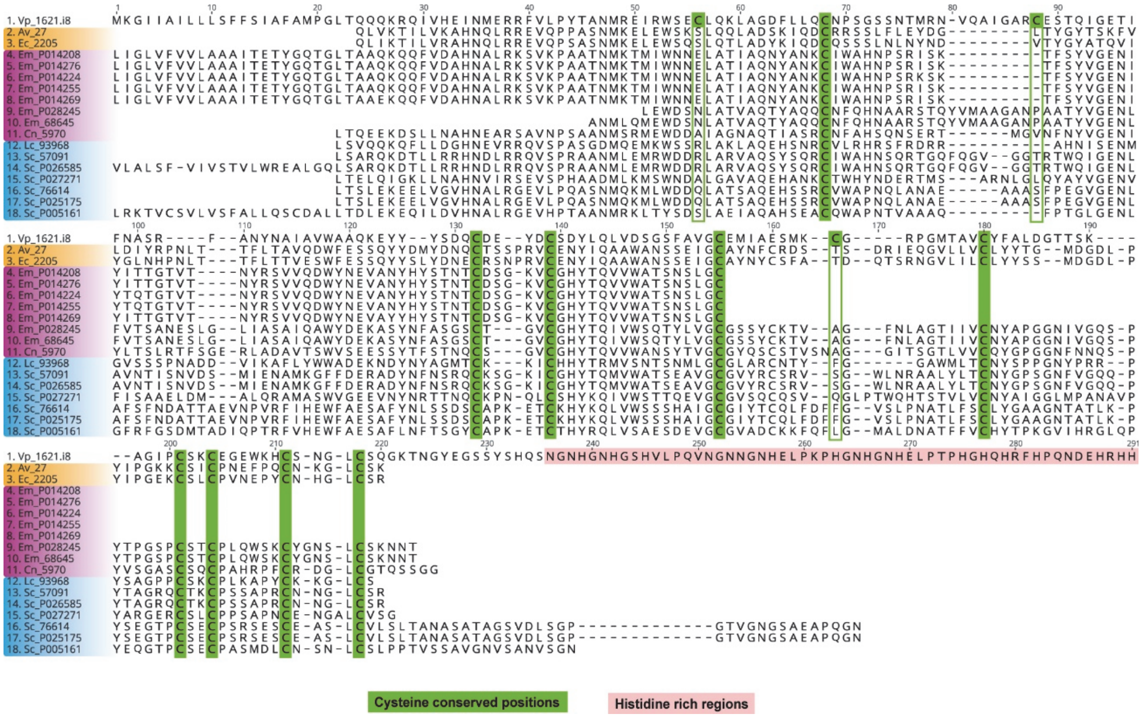


**Supplementary Fig. 9. Alignments of perisilin-like sequences of Porifera**. Conserved cysteine and histidine positions in perisilin-like proteins from Calcarea (blue), Demospongiae (purple) and Hexactinellida (orange) are compared to perisilin-1 of *Vazella pourtalesii* (Vp_1621.i8). The sequences were aligned using MAFFT-online. Sequence codes as in Fig. 7, Supplementary Fig. 10 and Supplementary Data 5. Alignment data is available in fasta format in Source Data Supplementary Fig. 9. All perisilin-like sequences show nine cysteine conserved positions out of the twelve cysteine conserved positions occurring in hexactinellid perisilins. No histidine-rich region related to silicification is present in any perisilin-like sequence of Calcarea, Demospongiae or Hexactinellida.

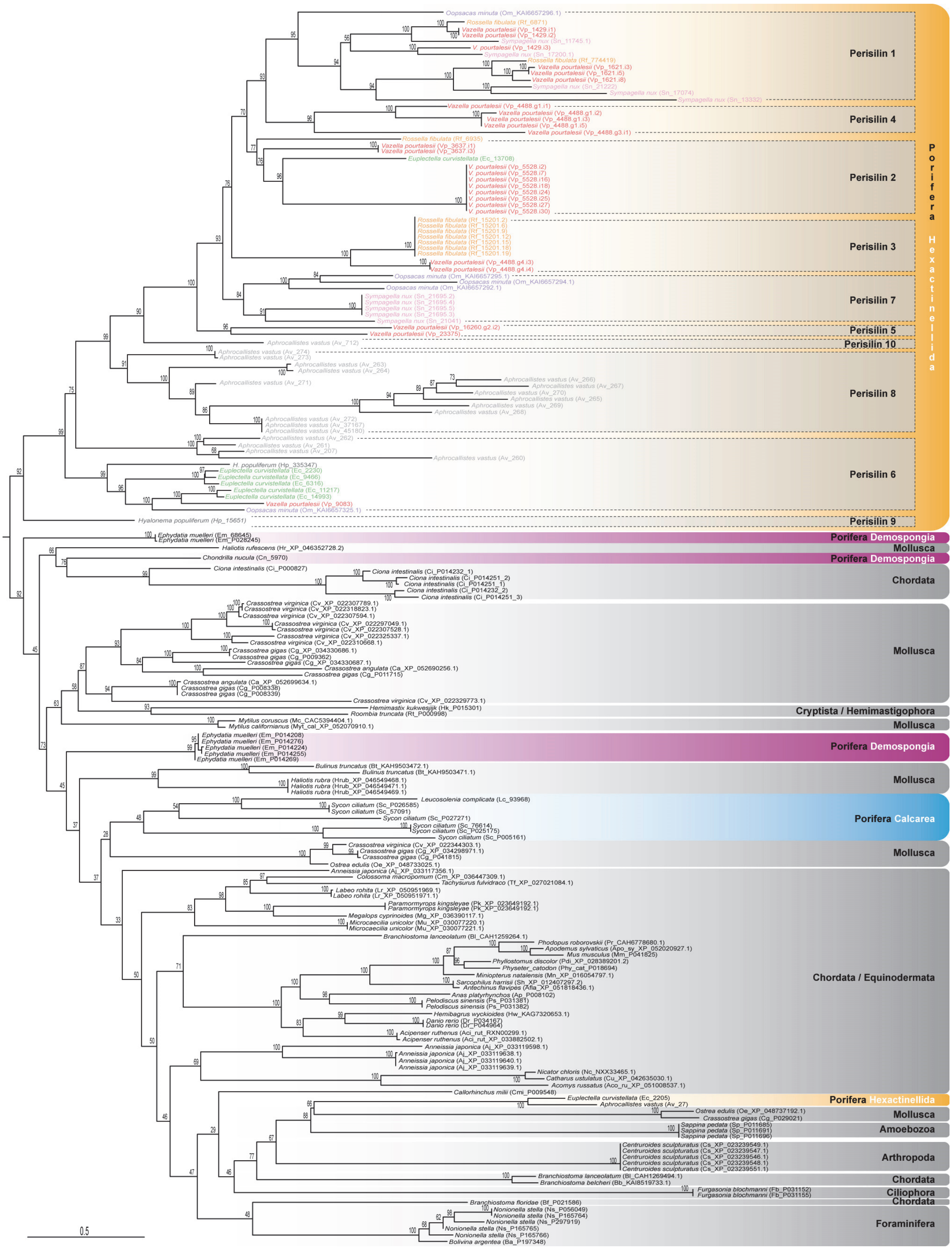


**Supplementary Fig. 10. Maximum Likelihood phylogenetic tree of perisilins obtained with IQ-TREE.** Included sequences correspond to all blast hits for perisilin-1α of *V. pourtalesii* (sequence Vp_1621.i8) with a bit score larger than 50 (Supplementary Data 5). Each hexactinellid species is represented by a different colour. Scale bar represents 0.4 amino acid substitutions per site. Bootstrap values are given at each node. Alignment data is available in fasta format in Source Data Fig. 7.

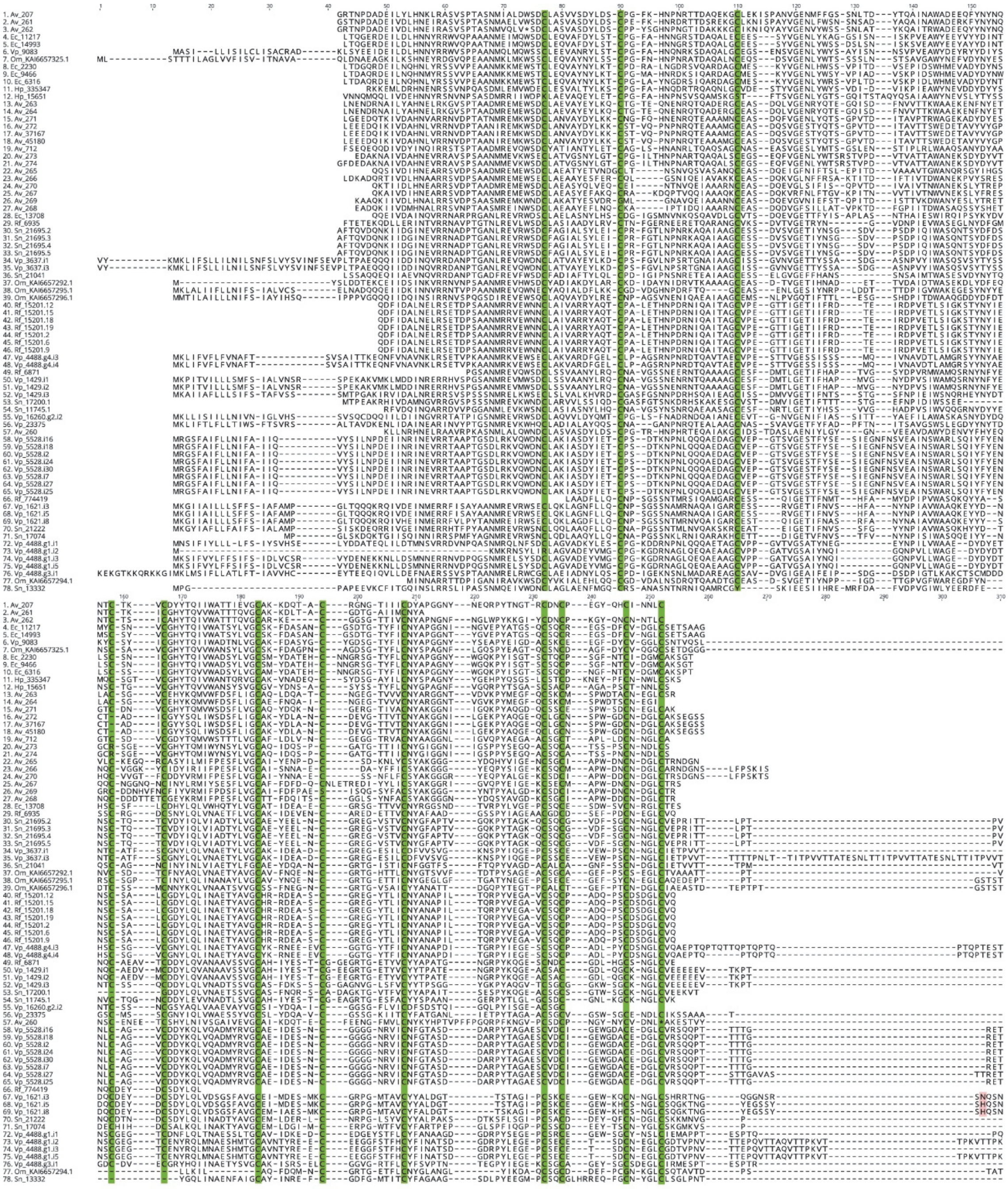

Cysteine conserved positions

Histidine rich regions

(Continued in the next page)

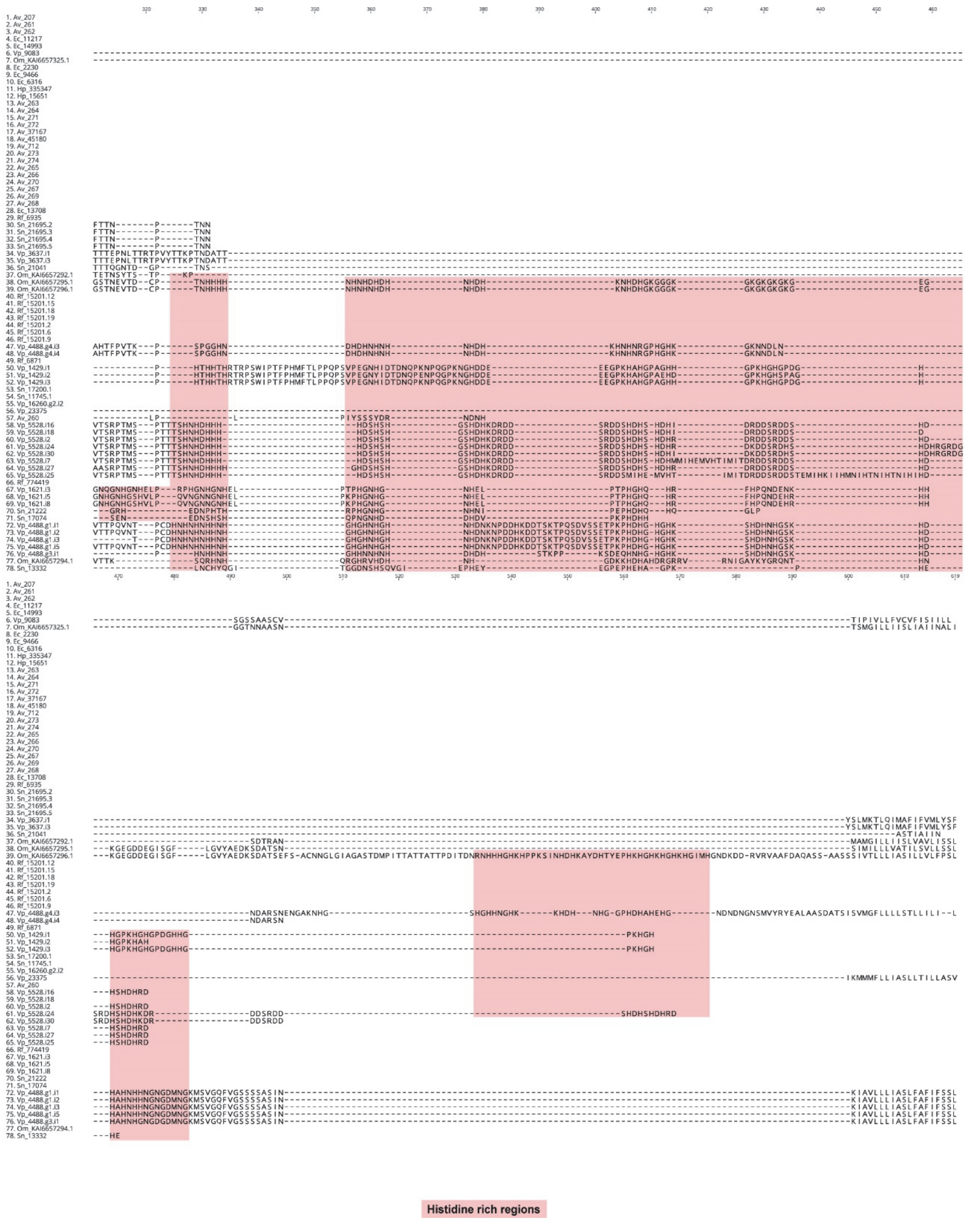

Supplementary Fig. 11. Conserved cysteine and histidine positions in hexactinellid perisilin proteins.

**Supplementary Fig. 11. Conserved cysteine and histidine positions in hexactinellid perisilin proteins.** All amino acid sequences of hexactinellid perisilins were aligned using MAFFT-online. Sequence codes as in Fig. 7, Supplementary Fig. 9 and Supplementary Data 5. Alignment data is available in fasta format in Source Data Fig. 7. Ec: *Euplectella curvistellata*, Av: *Aphrocallistes vastus,* Sn: *Sympagella nux*, Om: *Oopsacas minuta*, Rf: *Rossella fibulata*, Vp: *Vazella pourtalesii* and Hp: *Hyalonema populiferum.*

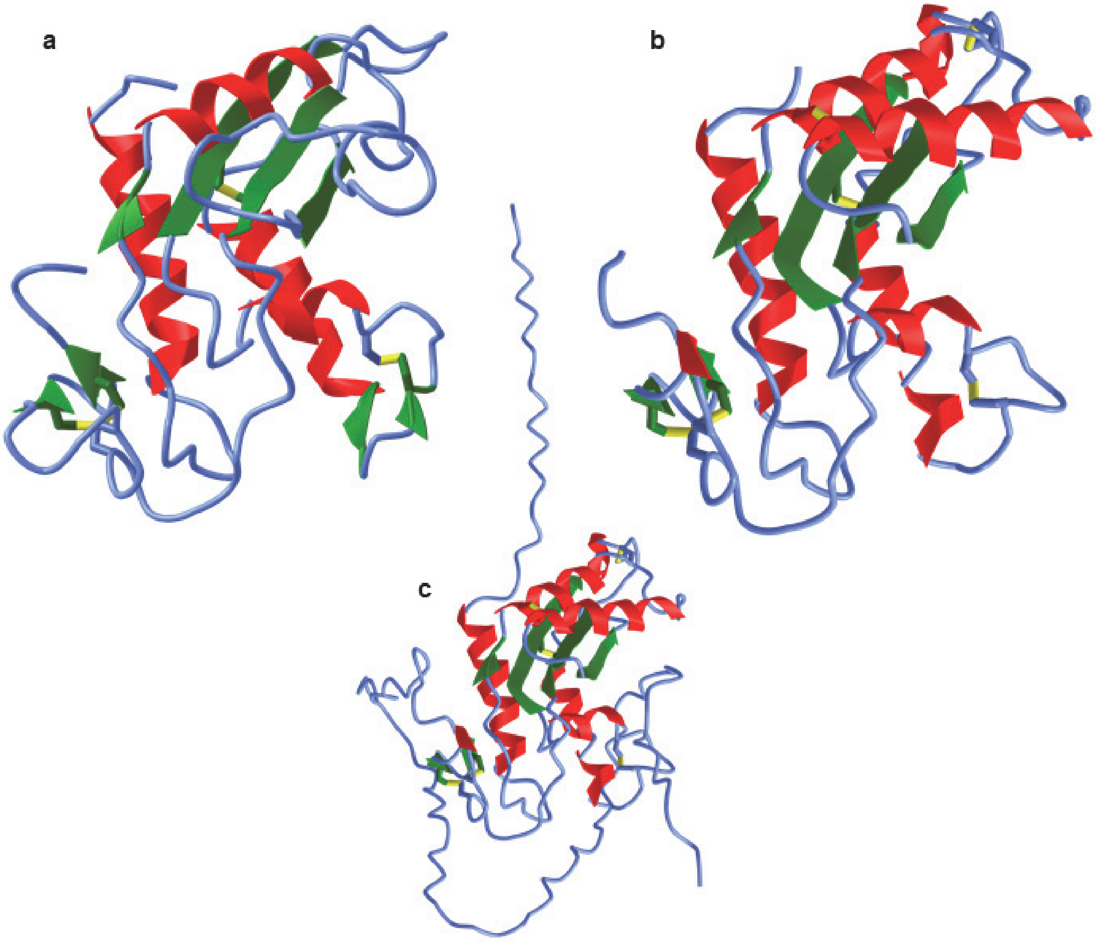


**Supplementary Fig. 12. 3D models of perisilin-1 of *Vazella pourtalesii.*** Models were built using 13q2u.1.A (Glioma pathogenesis-related protein 1) as a template for SWISS-MODEL **(a)** or Alphafold2 **(b, c)**. Model in image **a** represents amino acids 21-197 in the sequence, because the template covered only such a region (see Supplementary Fig. 6). The same peptide sequence (i.e., 21-197 aa) was selected for comparing 3D inference in image **b** using Alphafold2 (**b**). Three-D structure inferred by Alphafold2 on the basis of the entire aa sequence (**c**). Note that regions unavailable in the template are basically predicted by Alphafold2 as with random structure. Sequences expected to form alpha-helices and beta-sheets are colored in red and green, respectively.

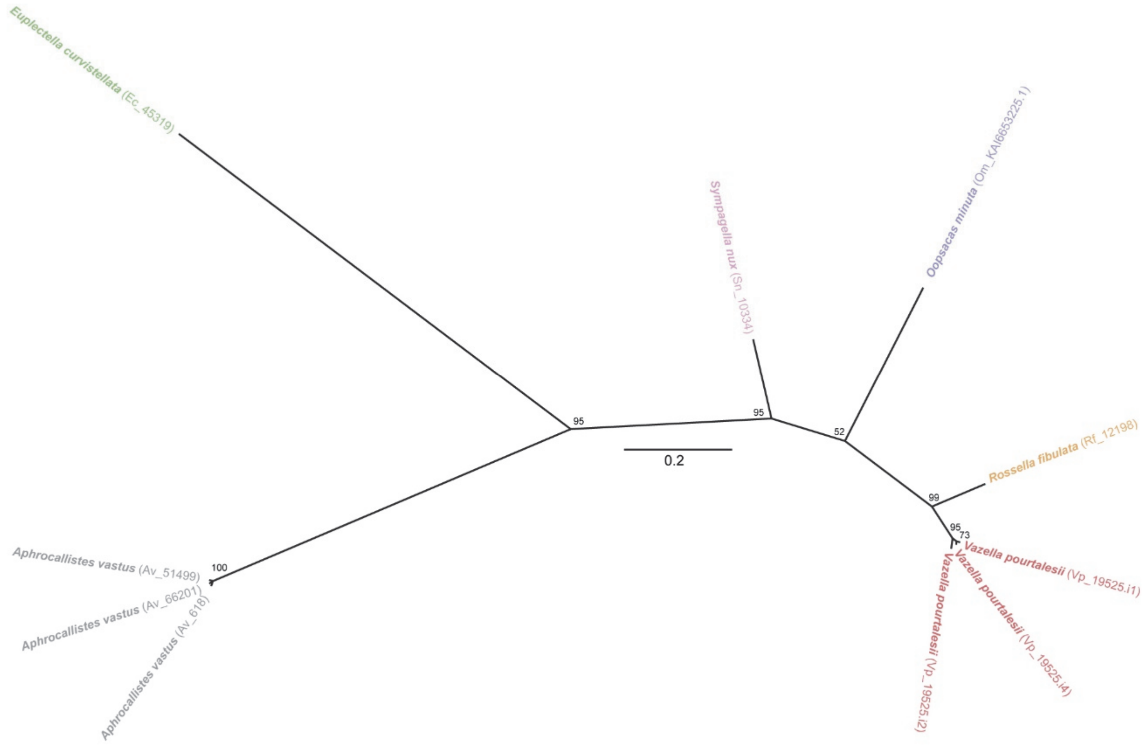


**Supplementary Fig. 13.** Unrooted Maximum Likelihood phylogenetic tree of glassins obtained with IQ-TREE. Included sequences correspond to all blast hits for glassin of *E. curvistellata* (sequence Ec_45319) with a bit score larger than 50 (Supplementary Data 7). Each hexactinellid species is represented by a different colour. Scale bar represents 0.2 amino acid substitutions per site. Bootstrap values are given at each node. Alignment data is available in fasta format in Source Data Fig. 8.

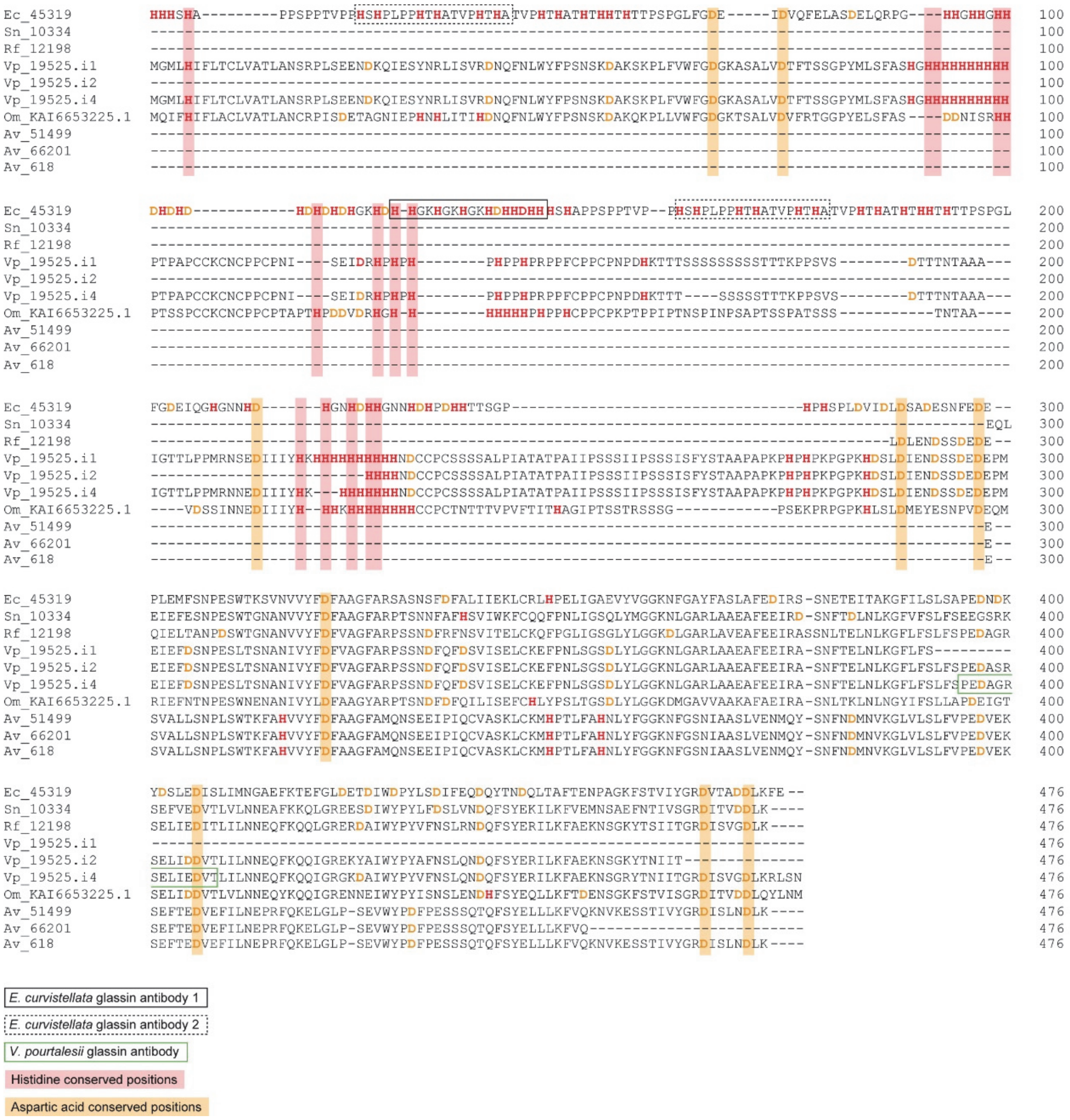


**Supplementary Fig. 14. Conserved histidine and aspartic acid positions in hexactinellid glassin proteins.** All amino acid sequences of hexactinellid glassins were aligned using MAFFT- online. Sequence regions in boxes correspond to the antibodies used for the immunodetection of glassin in *E. curvistellata* and *V. pourtalesii*. Sequence codes as in Fig. 8, Supplementary Fig. 13 and Supplementary Data 7. Ec: *Euplectella curvistellata*, Av: *Aphrocallistes vastus,* Sn: *Sympagella nux*, Om: *Oopsacas minuta*, Rf: *Rossella fibulata*, Vp: *Vazella pourtalesii* and Hp: *Hyalonema populiferum*.

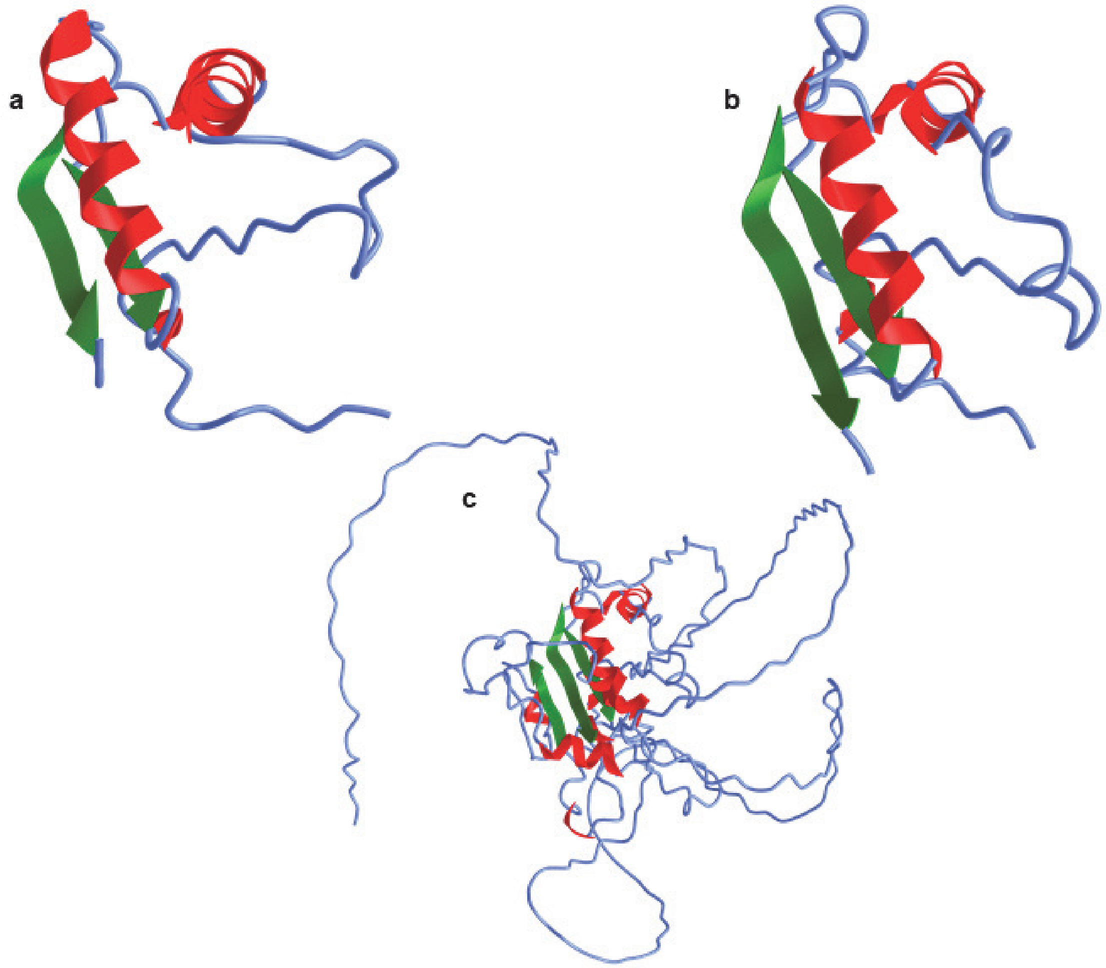


**Supplementary Fig. 15. 3D models of glassin of *Euplectella curvistellata*.** Models were built using 1wht.1.A (Serine carbopeptidase II) as a template for SWISS-MODEL **(a)** or Alphafold2 **(b, c)**. Model in image **a** represents amino acids 227-336 in the sequence, because the template covered only such a region (see Supplementary Fig. 6). The same peptide sequence (i.e., 227-336 aa) was selected for comparing 3D inference in image **b** using Alphafold2 (**b**). Three-D structure inferred by Alphafold2 on the basis of the entire aa sequence (**c**). Note that regions unavailable in the template are basically predicted by Alphafold2 as with random structure. Sequences expected to form alpha-helices and beta-sheets are colored in red and green, respectively.

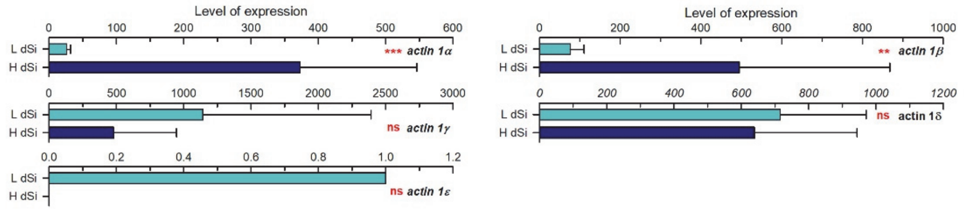


**Fig. 16. Expression pattern of actin in *Vazella pourtalesii*.** Average (±SD) TMM normalized expression values of *actin* genes in the transcriptome of six individuals of *V. pourtalesii* exposed to naturally low dSi concentrations (light blue) versus that of six others exposed to high dSi concentrations (dark blue). Asterisks indicate differentially expressed (DE) genes with statistical significance, following the criterion of at least fourfold expression and with the P-values corrected by false discovery rate (FDR): ***= P < 0.001; **= P < 0.01; ns= not significant. TMM expression values per individual and statistical tests are detailed in Supplementary Data 3-4.

# Supplementary Tables

**Supplementary Table S1.** Signal peptide and topology prediction of hexaxilin, perisilin and glassin obtained with the software Phobius.

| | **Hexaxilin-1 of *E. curvistellata* (Ec_20423)** | **Perisilin-1 of *V. pourtalesii* (Vp_1621.i8)** | **Glassin of *V. pourtalesii* (Vp_19525.i4)** |
|---|---|---|---|
| **Signal region** | 1 - 19 aa | 1 - 18 aa | 1 - 18 aa |
| N-region | 1 - 4 aa | 1 - 2 aa | 1 - 3 aa |
| H-region | 5 - 15 aa | 3 - 13 aa | 4 - 13 aa |
| C-region | 16 - 19 aa | 14 - 18 aa | 14 - 18 aa |
| **Non-cytoplasmic domain** | 20 - 359 aa | 19 - 265 aa | 19 - 445 aa |

# Supplementary References


1. Ciriminna, R. *et al.* The sol-gel route to advanced silica-based materials and recent applications. *Chem. Rev.* **113**, 6592–6620 (2013).
2. Wang, Y. *et al.* Mesoporous silica nanoparticles in drug delivery and biomedical applications. *Nanomedicine Nanotechnology, Biol. Med.* **11**, 313–327 (2015).
3. Ki, M. R., Nguyen, T. K. M., Jun, H. S. & Pack, S. P. Biosilica-enveloped ferritin cage for more efficient drug deliveries. *Process Biochem.* **68**, 182–189 (2018).
4. Betancor, L. & Luckarift, H. R. Bioinspired enzyme encapsulation for biocatalysis. *Trends Biotechnol.* **26**, 566–572 (2008).
5. Wang, G. *et al.* Hydrated silica exterior produced by biomimetic silicification confers viral vaccine heat-resistance. *ACS Nano* **9**, 799–808 (2015).
6. Müller, W. E. G. *et al.* Development of a morphogenetically active scaffold for three-dimensional growth of bone cells: Biosilica-alginate hydrogel for SaOS-2 cell cultivation. *J. Tissue Eng. Regen. Med.* **9**, E39–E50 (2015).
7. Wiens, M. *et al.* The role of biosilica in the osteoprotegerin/RANKL ratio in human osteoblast-like cells. *Biomaterials* **31**, 7716–7725 (2010).
8. Müller, W. E. G. *et al.* Biosilica-loaded poly(ε-caprolactone) nanofibers mats provide a

morphogenetically active surface scaffold for the growth and mineralization of the osteoclast-related SaOS-2 cells. *Biotechnol. J.* **9**, 1312–1321 (2014).
9. Ki, M.-R., Park, K. S., Abdelhamid, M. A. A. & Pack, S. P. Novel silicatein-like protein for biosilica production from *Amphimedon queenslandica* and its use in osteogenic composite fabrication. *Korean J. Chem. Eng.* **40**, 419–428 (2023).
10. Guo, J. *et al.* Multiscale design and synthesis of biomimetic gradient protein/biosilica composites for interfacial tissue engineering. *Biomaterials* **145**, 44–55 (2017).
11. Guo, J. *et al.* Coding cell micropatterns through peptide inkjet printing for arbitrary biomineralized architectures. *Adv. Funct. Mater.* **28**, 1800228 (2018).
12. Talevski, T. *et al.* Identification and first insights into the structure of chitin from the endemic freshwater demosponge Ochridaspongia rotunda (Arndt, 1937). *Int. J. Biol. Macromol.* **162**, 1187–1194 (2020).
13. Ehrlich, H. & Worch, H. Sponges as natural composites: from biomimetic potential to development of new biomaterials. in *Porifera Research. Biodiversity, Innovation and Sustainability* (eds. Custódio, M. R., Lôbo-Hajdu, G., Hajdu, E. & Muricy, G.) vol. 28 303–312 (Museu Nacional Rio de Janeiro, 2007).
14. Ehrlich, H. *et al.* First evidence of the presence of chitin in skeletons of marine sponges. Part II. Glass sponges (Hexactinellida: Porifera). *J. Exp. Zool. Part B Mol. Dev. Evol.* **308**, 473–483 (2007).
15. Ehrlich, H. *et al.* Nanostructural organization of naturally occurring composites-Part II: Silica-chitin-based biocomposites. *J. Nanomater.* **2008**, (2008).
16. Tabachnick, K. R., Menshenina, L. L., Pisera, A. & Ehrlich, H. Revision of *Aspidoscopulia* Reiswig, 2002 (Porifera: Hexactinellida: Farreidae) with description of two new species. *Zootaxa* **2883**, 1–22 (2011).
17. Ehrlich, H. *et al.* Supercontinuum generation in naturally occurring glass sponges spicules. *Adv. Opt. Mater.* **4**, 1608–1613 (2016).
18. Maldonado, M. *et al.* On the dissolution of sponge silica: Assessing variability and biogeochemical implications. *Front. Mar. Sci.* **9**, 2487 (2022).
19. Maldonado, M. *et al.* Siliceous sponges as a silicon sink: An overlooked aspect of benthopelagic coupling in the marine silicon cycle. *Limnol. Oceanogr.* **50**, 799–809 (2005).
20. Pile, A. J. & Young, C. M. The natural diet of a hexactinellid sponge: Benthic-pelagic coupling in a deep-sea microbial food web. *Deep. Res. Part I Oceanogr. Res. Pap.* **53**, 1148–1156 (2006).
21. Ehrlich, H. *et al.* Arrested in glass: Actin within sophisticated architectures of biosilica in sponges. *Adv. Sci.* 2105059 (2022) doi:10.1002/advs.202105059.
22. Ehrlich, H. Actin and the realization of unique biosilica-based architectures in sponges. in *11th World Sponge Conference. Book of Abstracts* (eds. van der Wind, N., Paix, B., de Voog, N. J. & Hrab, J.) 247 (Naturalis Biodiversity Center, 2022).
23. Michels, J., Vogt, J., Simon, P. & Gorb, S. N. New insights into the complex architecture of siliceous copepod teeth. *Zoology* **118**, 141–146 (2015).
24. Mann, S., Perry, C. C. & Webb, J. Structure, morphology, composition and organization of biogenic minerals in limpet teeth. *Proc. R. Soc. London - Biol. Sci.* **227**, 179–190 (1986).
25. Krings, W., Wägele, H., Neumann, C. & Gorb, S. N. Coping with abrasive food: diverging composition of radular teeth in two Porifera-consuming nudibranch species (Mollusca, Gastropoda). *J. R. Soc. Interface* **20**, 20220927 (2023).
26. Lu, S. *et al.* CDD/SPARCLE: the conserved domain database in 2020. *Nucleic Acids Res.* **48**, D265–D268 (2020).
27. Aravind, L. & Ponting, C. P. The cytoplasmic helical linker domain of receptor histidine kinase and methyl-accepting proteins is common to many prokaryotic signalling proteins. *FEMS Microbiol. Lett.* **176**, 111–116 (1999).
28. Baker, P. *et al.* Exopolysaccharide biosynthetic glycoside hydrolases can be utilized to disrupt and prevent *Pseudomonas aeruginosa* biofilms. *Sci. Adv.* **2**, (2016).
29. Sandford, F. Physical and chemical analysis of the siliceous skeletons in six sponges of

two groups (demospongiae and hexactinellida). *Microsc. Res. Tech.* **62**, 336–355 (2003).
30. Garrone, R., Simpson, T. L., Pottu-Boumendil, J. & Volcani, B. E. Ultrastructure and deposition of silica in sponges. in *Silicon and siliceous structures in biological systems* 495–550 (Springer-Verlag, 1981).
31. Reiswig, H. M. The axial symmetry of sponges spicules and its phylogenetic significance. *Cah. Biol. Mar.* **12**, 505–514 (1971).
32. Croce, G. *et al.* Fiber diffraction study of spicules from marine sponges. *Microsc. Res. Tech.* **62**, 378–381 (2003).
33. Croce, G. *et al.* Structural characterization of siliceous spicules from marine sponges. *Biophys. J.* **86**, 526–534 (2004).
34. Zlotnikov, I., Masic, A., Dauphin, Y., Fratzl, P. & Zolotoyabko, E. Composition and mechanical properties of a protein/silica hybrid material forming the micron-thick axial filament in the spicules of marine sponges. *Adv. Eng. Mater.* **16**, 1073–1077 (2014).
35. Gibbs, G. M., Roelants, K. & O'Bryan, M. K. The CAP superfamily: Cysteine-rich secretory proteins, antigen 5, and pathogenesis-related 1 proteins - Roles in reproduction, cancer, and immune defense. *Endocr. Rev.* **29**, 865–897 (2008).
36. Yamazaki, Y. & Morita, T. Structure and function of snake venom cysteine-rich secretory proteins. *Toxicon* **44**, 227–231 (2004).
37. Murphy, E. V., Zhang, Y., Zhu, W. & Biggs, J. The human glioma pathogenesis-related protein is structurally related to plant pathogenesis-related proteins and its gene is expressed specifically in brain tumors. *Gene* **159**, 131–135 (1995).
38. Asojo, O. A., Koski, R. A. & Bonafé, N. Structural studies of human glioma pathogenesis-related protein 1. *urn:issn:0907-4449* **67**, 847–855 (2011).
39. Imsiecke, G. & Müller, W. E. Unusual presence and intracellular storage of silica crystals in the freshwater sponges *Ephydatia muelleri* and *Spongilla lacustris* (Porifera: Spongillidae). *Cell. Mol. Biol.* **41**, 827–832 (1995).
40. Custodio, M. R., Hajdu, E. & Muricy, G. In vivo study of microsclere formation in sponges of the genus Mycale (Demospongiae, Poecilosclerida). *Zoomorphology* **121**, 203–211 (2002).
41. Mohri, K., Nakatsukasa, M., Masuda, Y., Agata, K. & Funayama, N. Toward understanding the morphogenesis of siliceous spicules in freshwater sponge: Differential mRNA expression of spicule-type-specific silicatein genes in *Ephydatia fluviatilis*. *Dev. Dyn.* **237**, 3024–3039 (2008).
42. Simpson, T. L. *The cell biology of sponges*. (Springer-Verlag, 1984).
43. Schröder, H. C., Wang, X., Tremel, W., Ushijima, H. & Muller, W. E. G. Biofabrication of biosilica-glass by living organisms. *Nat. Prod. Rep.* **25**, 455–474 (2008).
44. Wang, X. *et al.* Evagination of cells controls bio-silica formation and maturation during spicule formation in sponges. *PLoS One* **6**, e20523 (2011).
45. Müller, W. E. G. *et al.* Hierarchical composition of the axial filament from spicules of the siliceous sponge *Suberites domuncula*: From biosilica-synthesizing nanofibrils to structure- and morphology-guiding triangular stems. *Cell Tissue Res.* **351**, 49–58 (2013).
46. Uriz, M. J., Turon, X. & Becerro, M. A. Silica deposition in demosponges: spiculogenesis in *Crambe crambe*. *Cell Tissue Res.* **301**, 299–309 (2000).
47. Müller, W. E. G. *et al.* Formation of siliceous spicules in the marine demosponge *Suberites domuncula*. *Cell Tissue Res.* **321**, 285–297 (2005).
48. Schröder, H. C. *et al.* Co-expression and functional interaction of silicatein with galectin: matrix-guided formation of siliceous spicules in the marine demosponge *Suberites domuncula*. *J. Biol. Chem.* **281**, 12001–12009 (2006).
49. Wiens, M. *et al.* The role of the silicatein-[alpha] interactor silintaphin-1 in biomimetic biomineralization. *Biomaterials* **30**, 1648–1656 (2009).
50. Schloßmacher, U. *et al.* Silintaphin-1 – interaction with silicatein during structure-guiding bio-silica formation. *FEBS J.* **278**, 1145–1155 (2011).
51. Wang, X. & Müller, W. E. G. Complex structures – smart solutions: Formation of siliceous spicules. *Commun. Integr. Biol.* **4**, 684–688 (2011).
52. Gazave, E. *et al.* No longer Demospongiae: Homoscleromorpha formal nomination as a

fourth class of Porifera. in *Ancient Animals, New Challenges* 3–10 (Springer Netherlands, 2012). doi:10.1007/978-94-007-4688-6_2.
53. Muricy, G., Díaz, M. C., Hooper, J. N. A. & Van Soest, R. W. M. Order Homosclerophorida Dendy, 1905, Family Plakinidae Schulze, 1880. in *Systema Porifera. A guide to the classification of sponges* vol. 1 71–82 (Kluwer Academic / Plenum Publishers, 2002).
54. Wiedenmayer, F. Contributions to the knowledge of post-Paleozoic neritic and archibental sponges (Porifera). *Schweizerische Paläontologidche Abhandlungen* **116**, 1–147 (1994).
55. Reid, R. E. H. Mesozoic and Cenozoic choristid demosponges. in *Treatise on Invertebrate Paleontology. Part E: Porifera, Volume 3* (ed. Kaesler, R. L.) 175–197 (The Geological Society of America & The University of Kansas, 2004). doi:10.17161/dt.v0i0.5144.
56. Botting, J. P. & Muir, L. A. Spicule structure and affinities of the Late Ordovician hexactinellid-like sponge Cyathophycus loydelli from the Llanfawr Mudstones Lagerstätte, Wales. *Lethaia* **46**, 454–469 (2013).
57. Botting, J. P. & Muir, L. A. Early sponge evolution: A review and phylogenetic framework. *Palaeoworld* **27**, 1–29 (2018).
58. Fontorbe, G., Frings, P. J., De La Rocha, C. L., Hendry, K. R. & Conley, D. J. A silicon depleted North Atlantic since the Palaeogene: Evidence from sponge and radiolarian silicon isotopes. *Earth Planet. Sci. Lett.* **453**, 67–77 (2016).
59. Dohrmann, M. & Wörheide, G. Dating early animal evolution using phylogenomic data. *Sci. Reports 2017 71* **7**, 1–6 (2017).
60. Siever, R. Silica in the oceans: Biological-geochemical interplay. in *Scientists on Gaia* (eds. Scheneider, S. H. & Boston, P. J.) 287–295 (MIT Press, 1991).
61. Maldonado, M. *et al.* Sponge skeletons as an important sink of silicon in the global oceans. *Nat. Geosci.* **12**, 815–822 (2019).
