# Supplementary material for: Silica-associated proteins from hexactinellid sponges support an alternative evolutionary scenario for biomineralization in Porifera
Source: Nat Commun. 2024 Jan 7;15:181. doi: 10.1038/s41467-023-44226-7 (PMC10772126; doi:10.1038/s41467-023-44226-7)
Supplement: Supplementary file 3 — Description of Additional Supplementary Files [file 41467_2023_44226_MOESM3_ESM.pdf]

Description of Additional Supplementary Files

Supplementary Data 1. Blast results of the search for hexaxilin homologs. Sequence used as a query: Ec_20423 of E. curvistellata. Only hits with a bit score higher than 50 were retained.

Supplementary Data 2. Foldseek results of the search for hexaxilin homologs. Query: 3D secondary structure of Hexaxilin 1 of E. curvistellata (Ec_20423) inferred with Alphafold2.

Supplementary Data 3. Test for differential expression of hexaxilins, perisilins, glassin and actin between control and high dSi conditions in V. pourtalesii. Statistical test performed with edgeR (implemented in Trinity) using a four fould-change threshold. logFC: log-fold-change; logCPM: log counts per million; FDR: false discovery rate corrected p-value; Not available: the test could not be performed in these transcripts due to TMM values equal to zero in some of the conditions.

Supplementary Data 4. TMM expression values of hexaxilins, perisilins, glassins and actin in V. pourtalesii individuals experimentally exposed to low and high dissolved silicate concentrations (control and high dSi, respectively), with the average TMM value and standard deviation for each condition.

Supplementary Data 5. Blast results of the search for perisilin homologs. Sequence used as a query: Vp_1621.i8 of V. pourtalesii. Only hits with a bit score higher than 50 were retained.

Supplementary Data 6. Foldseek results of the search for perisilin homologs. Query: 3D secondary structure of Perisilin 1 of V. pourtalesii (Vp_1621.i8) inferred with Alphafold2.

Supplementary Data 7. Blast results of the search for glassin homologs. Sequence used as a query: Ec_45319 of E. curvistellata. Only hits with a bit score higher than 50 were retained.

Supplementary Data 8. GenBank Accession Numbers of hexaxilin, perisilin and glassin sequences of E. curvistellata and V. pourtalesii obtained in the present study.
