# Supplementary material for: Silica-associated proteins from hexactinellid sponges support an alternative evolutionary scenario for biomineralization in Porifera
Source: Nat Commun. 2024 Jan 7;15:181. doi: 10.1038/s41467-023-44226-7 (PMC10772126; doi:10.1038/s41467-023-44226-7)
Supplement: Supplementary file 12 — Reporting Summary [file 41467_2023_44226_MOESM12_ESM.pdf]

nature portfolio

Corresponding author(s): Manuel Maldonado & Katsuhiko Shimizu

Last updated by author(s): Nov 3, 2023

Reporting Summary

Nature Portfolio wishes to improve the reproducibility of the work that we publish. This form provides structure for consistency and transparency in reporting. For further information on Nature Portfolio policies, see our Editorial Policies and the Editorial Policy Checklist.

Statistics

For all statistical analyses, confirm that the following items are present in the figure legend, table legend, main text, or Methods section.

n/a	Confirmed	
☐	☒	The exact sample size (*n*) for each experimental group/condition, given as a discrete number and unit of measurement
☐	☒	A statement on whether measurements were taken from distinct samples or whether the same sample was measured repeatedly
☐	☒	The statistical test(s) used AND whether they are one- or two-sided<br>*Only common tests should be described solely by name; describe more complex techniques in the Methods section.*
☒	☐	A description of all covariates tested
☒	☐	A description of any assumptions or corrections, such as tests of normality and adjustment for multiple comparisons
☐	☒	A full description of the statistical parameters including central tendency (e.g. means) or other basic estimates (e.g. regression coefficient) AND variation (e.g. standard deviation) or associated estimates of uncertainty (e.g. confidence intervals)
☐	☒	For null hypothesis testing, the test statistic (e.g. *F*, *t*, *r*) with confidence intervals, effect sizes, degrees of freedom and *P* value noted<br>*Give P values as exact values whenever suitable.*
☐	☒	For Bayesian analysis, information on the choice of priors and Markov chain Monte Carlo settings
☒	☐	For hierarchical and complex designs, identification of the appropriate level for tests and full reporting of outcomes
☒	☐	Estimates of effect sizes (e.g. Cohen's *d*, Pearson's *r*), indicating how they were calculated

Our web collection on statistics for biologists contains articles on many of the points above.

Software and code

Policy information about availability of computer code

Data collection	No software or computer code was used to collect data.
Data analysis	SigmaPlot 14.5, NCBl-blast 2.12.0, EukProt v3, Trinity-v2.11.0, Kallisto, FigTree v1.3.1, MAFFT online v.7, IQ-TREE 1.6.0, MrBayes 3.2, FoldSeek web server, Phobius web server, Swiss-model, AlphaFold2 implemented in ColabFold-v1.5.2 platform, Adobe Illustrator 2023 v27.1.1, Adobe Photoshop, Geneious Prime 2022.0.1, Bioconductor package edgeR, R-v4.1.0, Trimmomatic-v0.39

For manuscripts utilizing custom algorithms or software that are central to the research but not yet described in published literature, software must be made available to editors and reviewers. We strongly encourage code deposition in a community repository (e.g. GitHub). See the Nature Portfolio guidelines for submitting code & software for further information.

Data

Policy information about availability of data

All manuscripts must include a data availability statement. This statement should provide the following information, where applicable:

- Accession codes, unique identifiers, or web links for publicly available datasets
- A description of any restrictions on data availability
- For clinical datasets or third party data, please ensure that the statement adheres to our policy

All sequence data generated in the present study, including the newly assembled transcriptome of Vazella pourtalesii, have been deposited in Figshare data repository (https://doi.org/10.6084/m9.figshare.23799351). Protein sequences are available at GenBank database, with accession codes and hyperlinks provided in Supplementary Data 8. Additionally, protein sequences are also available at the DNA Data Bank of Japan (https://www.ddbj.nig.ac.jp/index-e.html). The sources of

all previously published genomes and transcriptomes used for local blast searches are available as hyperlinks in Supplementary Data 1, 5, and 7. The unpublished genome of Euplectella curvistellata herein used is available at Tottori University repository https://repository.lib.tottori-u.ac.jp/records/7586. Raw transcriptomic reads of the twelve individuals of Vazella pourtalesii from a dSi-enrichment experiment published elsewhere15 were downloaded from Short Read Archive (SRA) under the BioProject number PRJNA580361. All quantitative and qualitative raw data supporting the findings and used for the statistical analyses are given in one Supplementary Table, eight files of Supplementary Data and seven files of Source Data provided with this paper.

Research involving human participants, their data, or biological material

Policy information about studies with human participants or human data. See also policy information about sex, gender (identity/presentation), and sexual orientation and race, ethnicity and racism.

Reporting on sex and gender	This study does not involve human researh
Reporting on race, ethnicity, or other socially relevant groupings	N/A
Population characteristics	N/A
Recruitment	N/A
Ethics oversight	N/A

Note that full information on the approval of the study protocol must also be provided in the manuscript.

Field-specific reporting

Please select the one below that is the best fit for your research. If you are not sure, read the appropriate sections before making your selection.

☒ Life sciences ☐ Behavioural & social sciences ☐ Ecological, evolutionary & environmental sciences

For a reference copy of the document with all sections, see nature.com/documents/nr-reporting-summary-flat.pdf

Life sciences study design

All studies must disclose on these points even when the disclosure is negative.

Sample size

Individuals of two hexactinellid sponge species (Vazella pourtalesii & Euplectella curvistellata) were selected at random from a large population to obtain spicules for extracting silicifying proteins and to conduct transcriptomic analysis. V. pourtalesii individuals were also used for transcriptomic and differential gene expression analyses. To these objectives, samples were collected as part of previous studies published elsewhere.
A group of 12 individuals of a large North-Atlantic population of the hexactinellid sponge Vazella pourtalesii were collected for transcriptomic analyses (6 controls + 6 treatments) as detailed in Maldonado et al. 2020: Maldonado, M., M. López-Acosta, L. Beazley, E. Kenchington, V. Koutsouveli, and A. Riesgo. 2020. “Cooperation between Passive and Active Silicon Transporters Clarifies the Ecophysiology and Evolution of Biosilicification in Sponges.” Science Advances 6(28):eaba9322.

Individuals of the North-Pacific hexactinellid Euplectella curvistellata were commercially purchased to fishermen and their spicules used to extract silicifying proteins as detailed in Shimizu et al. 2015: Shimizu, Katsuhiko, Taro Amano, Md. Rezaul Bari, James C. Weaver, Jiro Arima, and Nobuhiro Mori. 2015. “Glassin, a Histidine-Rich Protein from the Siliceous Skeletal System of the Marine Sponge Euplectella, Directs Silica Polycondensation.” Proceedings of the National Academy of Sciences, USA 112(37):11449–54.

Data exclusions

No data were excluded from the analyses. Results are presented considering all data cases without excluding outliers or any other form of departure from group average or centroids.

Replication

Transcriptomic analyses were based on a replication of 2 groups of 6 random individuals each. All attempts at replication were successful. Most other aspects of the study were not strictly concerned with replication, being basically searches in molecular databases and phylogenetic inference (conducted with high bootstrap replication).

Randomization

Allocation of individual and samples was random, except for transmision electron microscopy study, for which we selected individual showing high rates of silicate consumption.

Blinding

Blinding was not particularly needed because we were already blinded when attempting to extract from the sponge silica and sequencing novel proteins that were unannotated in genomes and transcriptomes.

Reporting for specific materials, systems and methods

We require information from authors about some types of materials, experimental systems and methods used in many studies. Here, indicate whether each material, system or method listed is relevant to your study. If you are not sure if a list item applies to your research, read the appropriate section before selecting a response.

Materials & experimental systems

n/a	Involved in the study
[ ]	[x] Antibodies
[x]	[ ] Eukaryotic cell lines
[x]	[ ] Palaeontology and archaeology
[x]	[ ] Animals and other organisms
[x]	[ ] Clinical data
[x]	[ ] Dual use research of concern
[x]	[ ] Plants

Methods

n/a	Involved in the study
[x]	[ ] ChIP-seq
[x]	[ ] Flow cytometry
[x]	[ ] MRI-based neuroimaging

Antibodies

Antibodies used

Pimary antibodies against proteins extrated from the silica are not commercially available. Rather author-designed, rabbit polyclonal antibodies against epitopes of proteins of interest were developed through Sigma-Aldrich Japan (Tokyo, Japan). For production of primary antibodies, short peptides (ranging from 13 to 20 aa) of the proteins of interest were selected as epitopes. For E. curvistellata, we selected the sequence CNSLEWLQEIKPQYAFSSNS, located at positions 253-272 of hexaxilin-1(Fig 2b) and the sequence HGKHGKHGKHDHHDHHH, located at positions 99 – 116 of glassin (Supplementary Fig. 14). For V. pourtalesii, we selected sequences AGTRSDYRDEAFQ (not successful) and CENDEVKHEMKAIPN (successfully working), located at positions 132-140 and 342-356 of hexaxilin-1, respectively (Fig. 2d), sequence NHGNHELPTPHGHQHRFHP, located at positions 239-257 of perisilin-1alpha (Fig. 2e), and sequence PEDAGRSELIEDVT, located at positions 364-377 of glassin transcript Vp_19525.i4 (Supplementary Fig. 14).

To detect glassin and hexaxilin of E. curvistellata on the gels and within the silica of the skeletal parts, the primary antibody against Ec glassin (1: 1,000 dilution) and Ec hexaxilin (1: 1,000 dilution) were used in the form of antisera. In contrast, antisera containing anti-hexaxilin and anti-perisilin of V. pourtalesii were affinity-purified with protein-G spin columns, and then used as primary antibodies (1: 100 dilution) for detection of Vp hexaxilin and Vp perisilin on gels, respectively. As also reported in previous literature, our antibodies performed similarly well, irrespective of being used in serum or affinity purified.

Two secondary antibodies were used as it follows:

1. For Westernblot, the secondary antibody was included in WesternBreeze Chromogenic Western Blot Immunodetection Kit, anti-rabbit (WB7105, Thermo Fisher Scientific, Waltham, MA, USA) and used following the protocol provided by the company.

2. For immunohistochemistry, Alexa 488 conjugated to goat anti-rabbit IgG (H+L) cross-adsorbed secondary antibody (A-11008, Thermo Fisher Scientific, Waltham, MA, USA) was used at the dilution of 1: 1,000.

Validation

We did not use any commercial primary antibody. Primary antibodies were all author-designed oned. Western blot analyses demonstrated that the primary antibodies recognized with specificity the target proteins (Supplementary Fig. 1). In the histochemical study, pre-immuned antisera were used as the negative control (Fig. 2).
